# Transient silencing of hypermutation preserves B cell affinity during clonal bursting

**DOI:** 10.1038/s41586-025-08687-8

**Published:** 2025-03-19

**Authors:** Juhee Pae, Niklas Schwan, Bertrand Ottino-Loffler, William S. DeWitt, Amar Garg, Juliana Bortolatto, Ashni A. Vora, Jin-Jie Shen, Alvaro Hobbs, Tiago B. R. Castro, Luka Mesin, Frederick A. Matsen, Michael Meyer-Hermann, Gabriel D. Victora

**Affiliations:** 1 Laboratory of Lymphocyte Dynamics, The Rockefeller University, New York, NY USA; 2 Department of Systems Immunology, Helmholtz Center for Infection Research, Braunschweig, Germany; 3 Center for Studies in Physics and Biology, The Rockefeller University, New York, NY USA; 4 Department of Genome Sciences, University of Washington, Seattle, WA USA; 5 Howard Hughes Medical Institute, New York, NY USA; 6 Computational Biology Program, Fred Hutchinson Cancer Research Center, Seattle, WA USA; 7 Howard Hughes Medical Institute, Seattle, WA USA; 8 Department of Statistics, University of Washington, Seattle, WA USA; 9 Lower Saxony Center for Artificial Intelligence and Causal Methods in Medicine (CAIMed), Hannover, Germany

**Keywords:** Germinal centres, Clonal selection, Computer modelling

## Abstract

In the course of antibody affinity maturation, germinal centre (GC) B cells mutate their immunoglobulin heavy- and light-chain genes in a process known as somatic hypermutation (SHM)^1–4^. Panels of mutant B cells with different binding affinities for antigens are then selected in a Darwinian manner, which leads to a progressive increase in affinity among the population^5^. As with any Darwinian process, rare gain-of-fitness mutations must be identified and common loss-of-fitness mutations avoided^6^. Progressive acquisition of mutations therefore poses a risk during large proliferative bursts^7^, when GC B cells undergo several cell cycles in the absence of affinity-based selection^8–13^. Using a combination of in vivo mouse experiments and mathematical modelling, here we show that GCs achieve this balance by strongly suppressing SHM during clonal-burst-type expansion, so that a large fraction of the progeny generated by these bursts does not deviate from their ancestral genotype. Intravital imaging and image-based cell sorting of a mouse strain carrying a reporter of cyclin-dependent kinase 2 (CDK2) activity showed that B cells that are actively undergoing proliferative bursts lack the transient CDK2^low^ ‘G0-like’ phase of the cell cycle in which SHM takes place. We propose a model in which inertially cycling B cells mostly delay SHM until the G0-like phase that follows their final round of division in the GC dark zone, thus maintaining affinity as they clonally expand in the absence of selection.

## Abstract (web summary)

B cell germinal centres achieve a balance between clonal expansion and sequence diversification by suppressing somatic hypermutation during proliferative clonal bursts.

## Main

The average rate at which germinal centre (GC) B cells accumulate mutations in their immunoglobulin (Ig) loci is estimated to be one per 1,000 bases per cell generation^14–16^, corresponding to about a two-thirds chance of acquiring at least one mutation per daughter cell when considering the roughly 700-bp length of the combined Ig heavy-chain (*Ighv*) and light-chain (*Igkv* or *Iglv*) variable regions. Because deleterious mutations—resulting in stop codons or reductions in antibody affinity or structural integrity—in general greatly outnumber affinity-enhancing mutations^6,17^, a crucial challenge for the GC is to identify multiple beneficial mutations while avoiding most, if not all, deleterious ones. Early mathematical models of GC mutation and selection proposed that a solution to this problem would be for cells to alternate phases of stochastic somatic hypermutation (SHM) and antigen-driven selection, the latter serving to purge the population of deleterious mutations while enriching for beneficial ones before allowing cells to mutate again. Assigning these two stages to the GC dark zone (DZ) and light zone (LZ), respectively, led to the establishment of the ‘cyclic re-entry’ paradigm, now the standard framework for how GC selection operates^11–13^.

More recently, we and others showed that the degree to which B cell clones expand varies considerably between individual GCs^7^ and with the amount of T cell help provided^8,10^. The fastest-growing clones undergo marked selective sweeps—which we termed clonal bursts—in which the descendants of a single B cell take over an entire 2,000-cell GC structure in a matter of days^7^. A large body of mechanistic work indicates that such bursts derive from B cells that received exceptionally strong proliferative signals from T follicular helper (T_FH_) cells present in the LZ. Help from T_FH_ cells triggers B cells to re-enter the DZ and undergo multiple rounds of proliferation by ‘inertia’ — that is, in the absence of further T cell-derived signals and thus in the absence of affinity-based selection^8–10,18–22^. Because such bursts require multiple rounds of inertial cell division not interspersed with affinity-based selection, the expectation, given a uniform rate of mutation per cell cycle, is that bursting B cells would rapidly accumulate deleterious mutations that would lead to precipitous declines in antibody affinity and integrity^11–13^. Thus, if a constant rate of SHM is assumed, the existence of clonal bursts would be incompatible with cyclic re-entry.

**Fig. 1:**
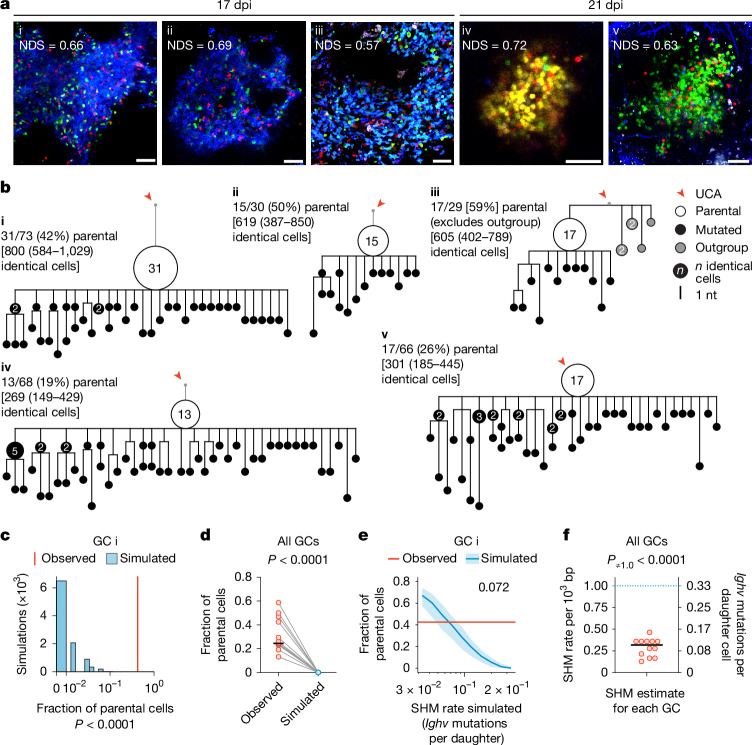
Clonal-burst phylogenies are incompatible with established GC SHM rates. **a**, Multiphoton images of single-coloured GCs indicative of clonal bursting, obtained at 17 or 21 dpi (10–14 days after the final dose of tamoxifen). The normalized dominance score (NDS) represents the fraction of cells belonging to the dominant colour, adjusted for fluorescent cell density^7^ (see Supplementary Data 1). Scale bars, 50 µm. **b**, Phylogenetic trees of *Ighv* sequences from the GCs in **a**. For each GC, the parental cell fraction and estimated counts of identical cells (±95% confidence interval (CI)), extrapolated to a 2,000-cell GC (in square brackets), are provided. Outgroup cells in GC iii are excluded from calculations. See additional trees in Extended Data Fig. 1b,c. nt, nucleotide; UCA, unmutated common ancestor. **c**, Observed parental fraction for GC i (red line) compared with distributions from 10,000 simulated clonal branching processes, matched to extrapolated GC sizes (blue bars). *P* values reflect the number of simulations with parental fractions at or above the observed value. **d**, Observed fractions of parental cells from 12 GCs (**b**,**c** and Extended Data Fig. 1b,c) compared with simulated fractions assuming a constant SHM rate of 0.333 *Ighv* mutations per daughter. *P* value by paired *t*-test. Horizontal black line shows the median. **e**, Median (±interquartile range) parental fractions from parameter sweep simulations of GC i with SHM rates increasing geometrically from 0.033 to 0.333 *Ighv* mutations per daughter (see Extended Data Fig. 2). The red line indicates the observed parental fraction for GC i. The number denotes the simulated SHM rate at which the median parental fraction most closely matched the observed fraction in vivo. **f**, Estimated SHM rates for 12 clonal bursts. *P* value by one-sample *t*-test against the theoretical SHM rate of 0.333 *Ighv* mutations per daughter. Horizontal black line shows the median.

**Extended Data Fig. 1:**
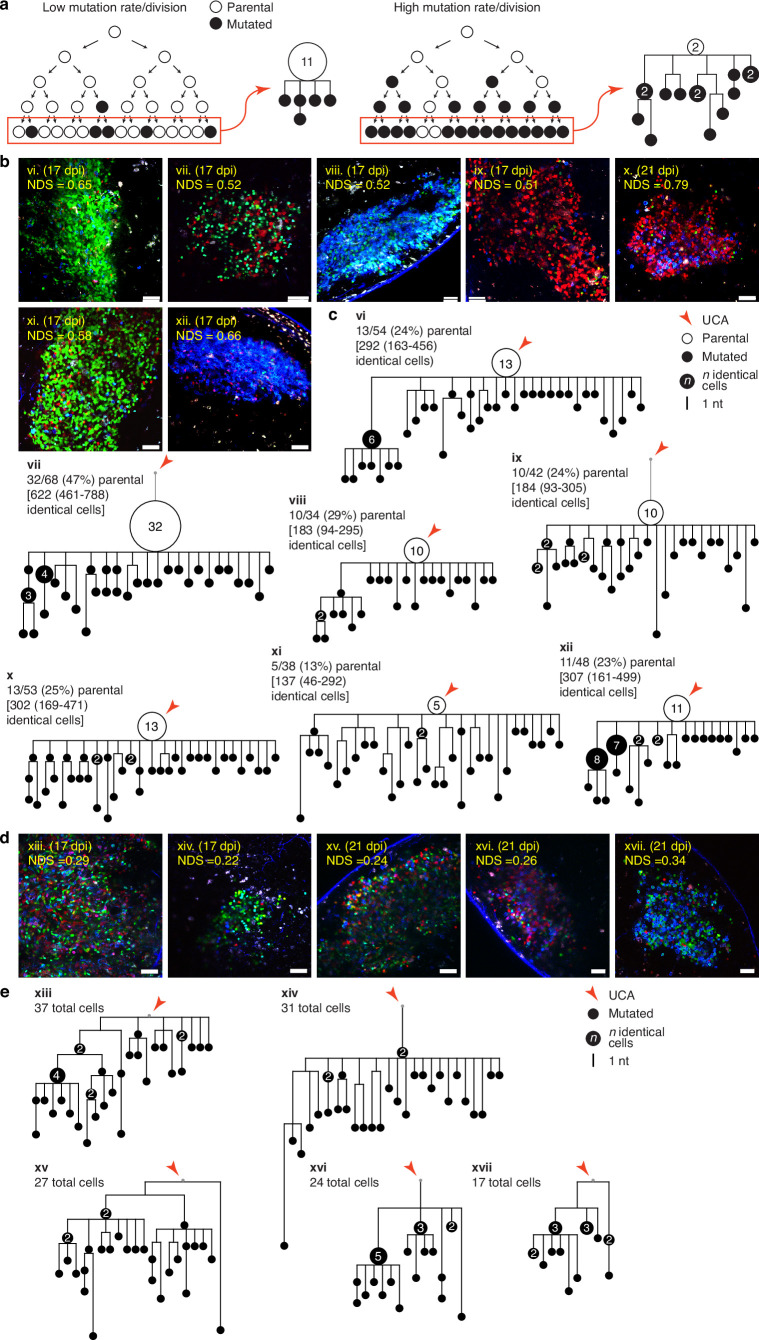
Phylogenies of B cell clones from additional bursting and non-bursting GCs. **a**, Illustration of GC B cell pedigrees assuming high or low SHM rates and their conversion into phylogenetic tree structures. Low rates generate larger “parental” nodes containing multiple identical sequences. **b**, Multiphoton images of additional single-coloured GCs indicative of clonal bursting, scanned at 17 or 21 dpi (10–14 days after the final dose of tamoxifen). See also Supplementary Data 1 for calculations. NDS, normalized dominance score, representing the approximate fraction of cells in the GC belonging to the dominant colour, normalized by fluorescent cell density^7^. Scale bar = 50 µm. **c**, *Ighv* sequences obtained from the GCs in **a** were used to construct phylogenetic trees. For each GC, the fraction of parental cells and the estimated total number of identical cells (±95%CI) in the burst when extrapolated to a 2,000-cell GC (in square brackets) are given. **d**, Multiphoton images of multicoloured GCs suggestive of higher B cell lineage diversity scanned at 17 or 21 dpi (10 or 14 days after the final dose of tamoxifen), details as in **a**. Scale bar = 50 µm. **e**, Phylogenetic trees as in **c**, but parental clones are not indicated, given that they are not defined in non-bursting lineages. GC xiii. was obtained from the same lymph node as GC i. (Fig. 1a) and the dominant lineage shown is an expansion of the same B cell clone (same V_H_/J_H_, and CDR_H_3 length) that was expanded in GC i. (Fig. 1b).

To resolve this apparent conflict, we began by examining the mutational patterns of clonal bursts at single-GC resolution. To this end, we used *Aicda*^CreERT2/+^.*Rosa26*^Confetti/Confetti^ (AID-Brainbow) mice^7,23,24^, in which multicolour fate-mapping of a Brainbow^25^ allele in B cells expressing activation-induced cytidine deaminase (AID) allows for easy identification of GCs containing large clonal bursts by microscopy. We immunized these mice with the model antigen chicken IgY in alum adjuvant to form GCs, triggered Brainbow recombination by treatment with tamoxifen starting on day 5 post-immunization (dpi) and scanned popliteal lymph nodes (pLNs) collected at 17 or 21 dpi for single-coloured GCs with a normalized dominance score (NDS; a measure of the proportion of cells of the dominant colour within the GC) greater than 0.5, indicative of clonal-burst-like expansion^7,26^. Using single-GC microdissection^7^, we isolated B cells from 12 such GCs and sequenced their *Ighv* genes. We then used sequences belonging to the dominant clone in the GC to build mutational phylogenies in an unsupervised manner using the gctree algorithm^27^. In gctree phylogenies, B cells that fail to mutate while rapidly proliferating appear as large nodes containing multiple cells with an identical *Ighv* sequence, followed by a variable number of descending branches. The ratio of the abundance of B cells at the parental node (defined as the node with the greatest number of identical sequences in the bursting phylogeny, which probably represents the B cell that initiated the clonal burst) over the abundance of its mutated descendants is inversely related to the mutational probability per division (Extended Data Fig. 1a). Dominant phylogenies derived from the 12 sequenced clonal bursts (9 from 17 dpi and 3 from 21 dpi) consisted uniformly of structures with large parental nodes, with an average fraction of parental cells of 0.32 ± 0.14 (s.d.) (Fig. 1a,b and Extended Data Fig. 1b,c). By contrast, larger phylogenies derived from five control GCs with NDS < 0.3 (including one phylogeny (GC vi) that was obtained from the same lymph node as GC i and in which a sister lineage of the same B cell clone was expanded) were generally more branched and, although quantitative comparisons are not possible given that a parental node by definition cannot be assigned to such lineages, generally lacked nodes with a large number of identical cells (Extended Data Fig. 1d,e).

**Extended Data Fig. 2:**
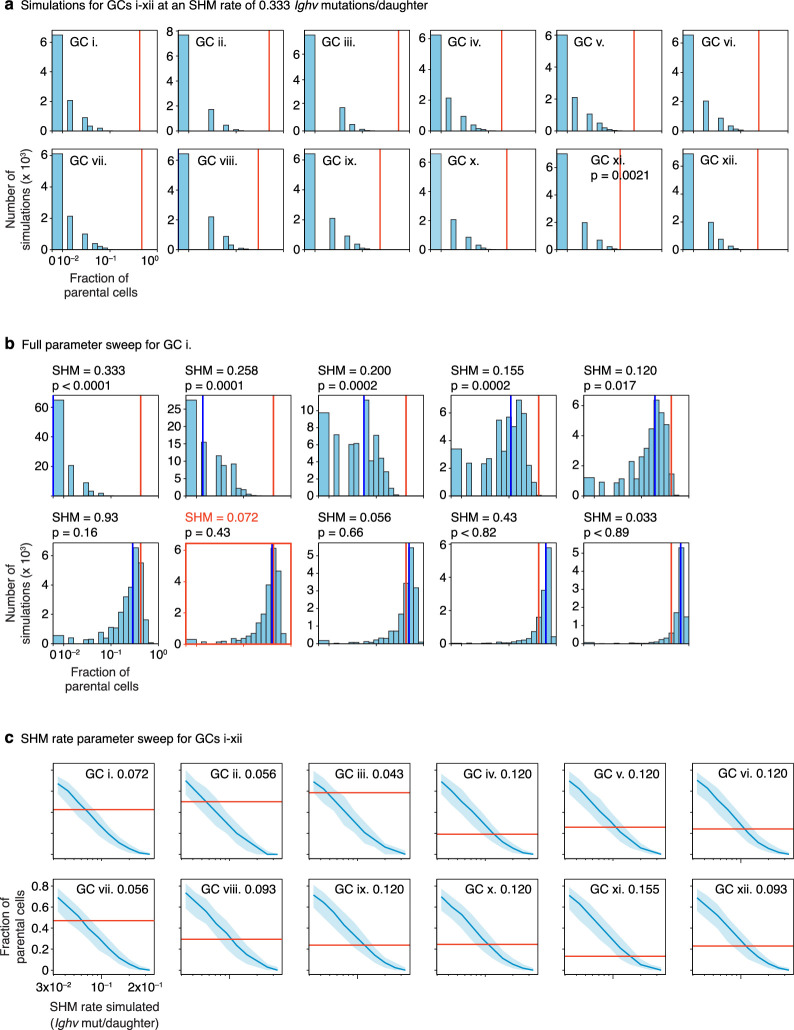
Brainbow GC simulations. **a**, Observed parental fractions for each of the 12 GCs in Fig. 1a,b and Extended Data Fig. 1b,c (red lines) compared to the distribution of the results of 10,000 simulations of clonal branching processes matched to the number of predicted cells in the experimental GC (blue bars). P values are calculated as the fraction of simulations with parental proportions at or above the observed value, and are <0.0001 unless otherwise indicated. **b**, Example of an SHM rate parameter sweep. Plots show results of iterative simulations of GC i. (Fig. 1b) where the SHM rate is decreased geometrically from 0.333 to 0.033. The SHM rate set for each set of simulations is given above the graph. The number of simulations run for each row decreases geometrically from 10,000 for the 0.333 to 1,000 for the 0.033 simulation, the first of which is reproduced from Fig. 1c. P values are given as the fraction of simulations that exceed the parental fraction of the observed GC (shown as a dotted red line). Median values for the set of simulations are shown as a blue line. The simulation whose median value most closely approaches the observed value for that GC is highlighted in red. **c**, Median (±interquartile range) parental fractions for parameter sweeps as in **b** for the entire 12-GC set in Fig. 1a,b and Extended Data Fig. 1b,c. The plot for GC i. is reproduced from Fig. 1e. The parental fraction of the observed GC is shown as a red line. Numbers indicate the simulated SHM rate with parental fraction closest to that observed for each GC.

Linear extrapolation of the measured fraction of parental cells to a 2,000-cell GC^7^ indicated that the clonal expansions in our 12 single-coloured GCs must contain on the order of several hundred B cells with identical *Ighv* sequences (Fig. 1b and Extended Data Fig. 1c). This is, at face value, incompatible with the premise of a constant mutation rate of 1 per 10^3^ bases per daughter cell^14–16^: assuming a uniform mutation rate and an approximately two-thirds chance of a descendant retaining the parental sequence (here we are looking at the *Ighv* segment only, which is close to 300 bp in length), the anticipated outcome after a 10-division burst is that, on average, only 18 (that is, 2/3^10^ × 2^10^) of 1,024 (2^10^) B cells would retain the parental sequence. To formally test this intuition, we used a birth–death process simulation^28^ to generate phylogenies with a constant *Ighv* mutation probability of one mutation per 1,000 bp incorporated (or one-third for each daughter after a cell division). For each GC in Fig. 1a, we ran simulations until the number of cells matched the empirical estimates from the sequenced clones (square brackets in Fig. 1b and Extended Data Fig. 1c). We performed 10,000 such simulations for each GC, randomly sampled cells from each simulation to match the number of cells found in the original in vivo phylogeny and then calculated the fraction of parental-type cells in each simulated sample (Fig. 1c,d and Extended Data Fig. 2a). In only 21 of the 120,000 simulation-derived samples did the parental fraction reach that observed in the original GCs in vivo. All 21 such instances were simulations of GC xi (Extended Data Fig. 1c), which had an exceptionally low fraction of parental cells and thus is likely to represent a burst that took place earlier in the course of the GC response. The median parental fraction for all simulations was zero, compared with 0.25 for experimentally measured GCs (Fig. 1d). A parameter sweep showed that the SHM rate that most closely approximated the observed parental fraction for each GC was, on average, 0.10 (range 0.043–0.16) mutations per *Ighv* region per daughter cell (equivalent to 0.30 (0.13–0.46) mutations per 10^3^ bases per generation; Fig. 1e,f and Extended Data Fig. 2b,c). Thus, clonal-burst phylogenies exhibit SHM rates that are between one-half and one-eighth of previously established average GC mutation rates^14–16^.

**Extended Data Fig. 3:**
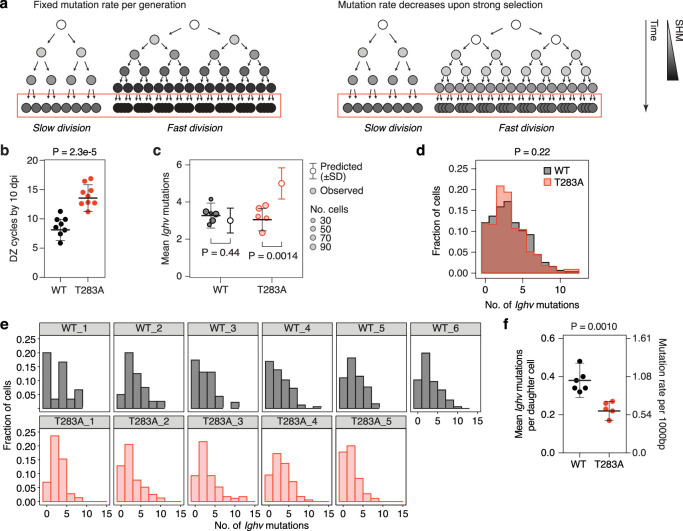
SHM rate decreases during inertial cell cycling in *Ccnd3*^*T283A*/+^ GC B cells. **a**, Schematic representation of SHM gain over time and division cycles in fixed (left) versus dynamic (right) SHM rate models. **b**, Predicted number of DZ inertial divisions by 10 dpi, based on previously reported data on cells entering the S-phase within the DZ for *Ccnd3*^+/+^ (WT) and *Ccnd3*^*T283A*/+^ (T283A) GC B cells^21^. Each symbol represents one mouse from 2 independent experiments. Predicted mean is inverse-variance-weighted and standard deviation accounts for variances within individual mice. P values are for Student’s t-test. **c**, Mean number of mutations per *Ighv* region for WT and T283A mice, compared to expected numbers assuming a uniform rate of division of 1 in 10^3^ bases per daughter cell. Predicted values were obtained as described in the methods section. Each symbol represents one mouse from 2 independent experiments. The symbol size represents the number of cells sequenced and analysed from each mouse. Predicted mean is inverse-variance-weighted and standard deviation accounts for variances within individual mice. P values are for Student’s t-test. **d**, Distribution of mutations in pooled WT and T283A cells from **c**. P values are for Student’s t-test. **e**, Distribution of mutations in GC B cells from individual WT and *Ccnd3*^*T283A*/+^ GC B cells. **f**, Inferred SHM rate for individual mice as in **c**. Each dot represents one mouse, inverse-variance-weighted mean and standard deviation is pooled to account for variances within individual mice. P values are for Student’s t-test.

Our findings raise the hypothesis that GC B cells actively downregulate SHM during clonal bursting. To test this, we sought to measure SHM in the presence or absence of perturbations that promote the inertial proliferation of GC B cells that drives bursting^21^ (Extended Data Fig. 3a). We first examined a genetic model in which GC B cells carry a Burkitt-lymphoma-associated gain-of-function mutation in the *Ccnd3* gene (*Ccnd3*^*T283A*^). This mutation slows the nuclear export and proteasomal degradation of the cell-cycle regulator cyclin D3, leading to moderate increases in DZ inertial cell cycling and in the size of the DZ compartment itself^21,29,30^. Previously, we used double-nucleotide pulsing^10^ to obtain the average percentages of wild-type (WT) and *Ccnd3*^*T283A*/+^ GC B cells entering S phase within the DZ in a 30-minute window (3.63% ± 0.26% (s.d.) and 5.80% ± 0.23%, respectively)^21^. On the basis of these data, we estimated that T283A-mutant GC B cells had undergone 67% more inertial cycles in the DZ by 10 dpi (13.5 versus 8.1) (Extended Data Fig. 3b). Assuming a uniform mutation rate per division cycle among DZ B cells, we would expect *Ccnd3*^*T283A*/+^ B cells to accumulate more mutations than WT cells (5.01 ± 0.84 versus 3.01 ± 0.67 mutations per cell, respectively) (Extended Data Fig. 3c). However, the observed SHM distributions in WT and *Ccnd3*^*T283A*/+^ GC B cells were indistinguishable (Extended Data Fig. 3c–e), and, accordingly, the estimated mutation rate of *Ccnd3*^*T283A*/+^ GC B cells was significantly lower than that of WT GC B cells (Extended Data Fig. 3f). More precisely, whereas mutations among WT cells occurred at a rate close to the predicted one^14–16^ (0.38 ± 0.09 mutations per daughter, or 1.01 ± 0.15 mutations per 10^3^ bases), mutations in *Ccnd3*^*T283A*/+^ GC B cells were substantially less frequent than expected (0.22 ± 0.05 mutations per daughter, or 0.59 ± 0.08 mutations per 10^3^ bases) (Extended Data Fig. 3f). Therefore, despite increasing the number of DZ cells engaging in inertial cycling, *Ccnd3*^*T283A*/+^ mice did not show a concomitant increase in mutation accrual over time, pointing to a reduced mutation rate per cell division.

**Fig. 2:**
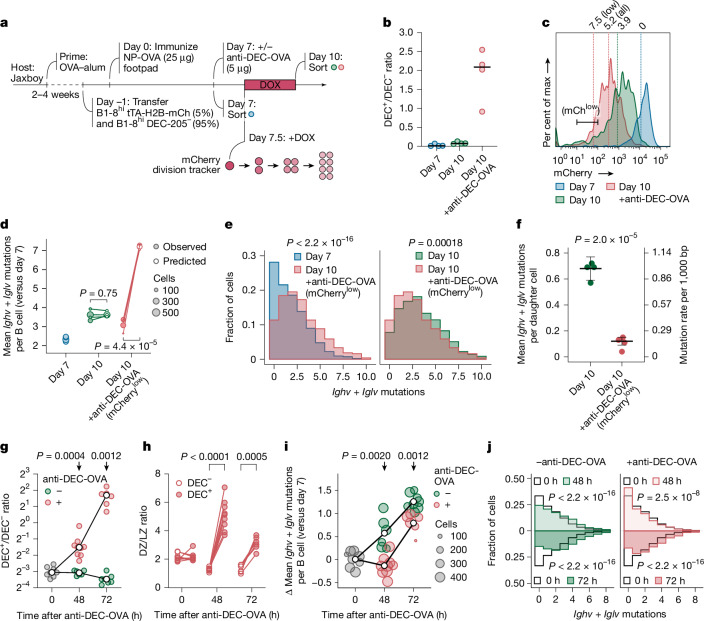
SHM rate decreases during inertial cell cycling in the DZ. **a**, Schematic of the anti-DEC-OVA experiment. **b**, Ratio of DEC-205^+^ to DEC-205^–^ B1-8^hi^ GC B cells in anti-DEC-OVA-treated and untreated mice. **c**, Flow-cytometry plot showing estimated number of cell divisions, as assessed by mCherry dilution (factor of 2 per generation), in B1-8^hi^ GC B cell populations. Estimated mean division numbers are shown above the histogram. DEC-205^+^ cells used in the analysis were sorted using the mCherry^low^ gate. **d**, Mean mutation counts per *Ighv* + *Iglv* region per mouse. Observed mutation counts are compared to predictions based on a uniform mutation rate of 1 in 1,000 bases per daughter cell. Each symbol represents one of four mice per group from two separate experiments. Horizontal black lines show the median. **e**, Mutation distributions in pooled samples from **d**. Data for individual mice are in Extended Data Fig. 4a. **f**, Inferred SHM rate for individual mice from **d**. Horizontal black lines indicate inverse variance-weighted mean with s.d. accounting for variances within individual mice. **g**, Ratio of DEC-205^+^ to DEC-205^–^ B1-8^hi^ GC B cells in anti-DEC-OVA-treated and untreated mice as in **a**. The white circle indicates the median for each group. Each symbol represents one of 6–9 mice per group from 2 independent experiments. **h**, DZ/LZ distribution of DEC-205^+^ and DEC-205^–^ B1-8^hi^ GC B cells in anti-DEC-OVA-treated mice. **i**, Mean change in mutation counts per *Ighv* + *Iglv* region in anti-DEC-OVA-treated and untreated mice, relative to the 0-h time-point mean. The white circle indicates the median for each group. **j**, Distribution of mutations in pooled 0-h, 48-h and 72-h samples from **i**. Data for individual mice are in Extended Data Fig. 4b. *P* values by Student’s *t*-test (**f**,**g**,**i**), Kolmogorov–Smirnov test (**e**,**j**), or paired *t*-test (**d**,**h**).

We next used a model in which we could deliberately force several B cell lineages to undergo multiple rounds of inertial cycling in the DZ en masse^8^ (Fig. 2a). We combined this experimental model with a genetically encoded tracker that measures cell division as a function of the dilution of mCherry fluorescent protein^10^. WT hosts received an adoptive transfer of B1-8^hi^ B cells (specific for 4-hydroxy-3-nitro-phenylacetyl; NP), of which 5% express mCherry under a doxycycline (DOX)-sensitive promoter, as well as the receptor DEC-205 (encoded by the gene *Ly75*), whereas the remaining 95% genetically lack DEC-205 expression (*Ly75*^–/–^). We then immunized these mice with NP coupled with ovalbumin (OVA) to generate GCs. Treating mice with established GCs with an antibody specific for DEC-205 coupled to OVA (anti-DEC-OVA) leads to enhanced presentation of OVA to T_FH_ cells by DEC-205-expressing GC B cells only, forcing these to interact with T_FH_ cells, migrate to the DZ and then rapidly undergo several rounds of proliferation in the absence of LZ selection^8–10^. We analysed GC B cells 72 h after treatment with anti-DEC-OVA (10 dpi), after cells have undergone substantial proliferative expansion in the DZ (Fig. 2b) but mostly before they have initiated a subsequent round of selection in the LZ.

**Extended Data Fig. 4:**
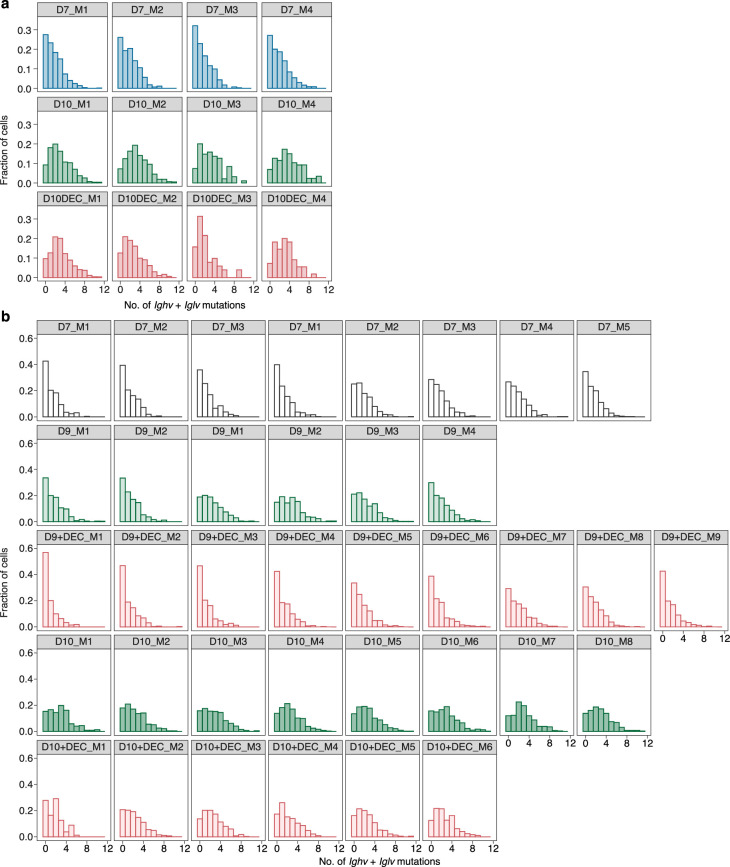
Mutation count data segregated by mouse. **a**, Distribution of mutations in GC B cells from individual mice at 7 dpi, 10 dpi untreated, and 10 dpi anti-DEC-OVA-treated DEC-205^+^ and DEC-205^–^ GC B cells as described in Fig. 2a–d. Aggregated data are shown in Fig. 2e. **b**, Distribution of mutations in DEC-205^+^ GC B cells from individual mice at 0, 48, and 72 h after treatment with anti-DEC-OVA, as well as respective non-treated controls. Aggregated data are shown in Fig. 2j.

Setting the mCherry fluorescence intensity measured at 7 dpi as the baseline, we calculated the extent of cell division from 7 to 10 dpi, with or without anti-DEC-OVA-induced proliferation. On average, cells underwent 3.9 divisions without the treatment and 5.2 divisions with the treatment (Fig. 2c). We then sorted B1-8^hi^ cells to directly measure SHM loads by droplet-based paired Ig sequencing. Specifically for the day-10 sample treated with anti-DEC-OVA, we sorted DEC-205-expressing B1-8^hi^ cells that had low levels of mCherry expression (corresponding to approximately 7.5 divisions, on average), to avoid confounding effects from lineages that underwent fewer divisions (Fig. 2c). Assuming a mutation rate of one per 1,000 bases for each daughter cell, we would expect to gain, by day 10, 3.5 and 9.3 *Ighv* + *Iglv* mutations (around 700 bases) in untreated and treated mice, respectively (Fig. 2d). Whereas the SHM accumulation from 7 to 10 dpi in GC B cells from untreated mice aligned with our predictions, averaging 1.6 mutations per cell, the empirical outcome for mice treated with anti-DEC-OVA was notably distinct. Not only did their GC B cells accumulate fewer mutations than predicted, but these cells also exhibited a slightly lower mutation count than their untreated counterparts (mean 1.1 mutations per cell; Fig. 2d,e and Extended Data Fig. 4a). Accounting for division rates, these mutational loads corresponded to a decrease in the SHM rate from 0.67 ± 0.10 *Ighv* + *Iglv* mutations per daughter cell (or 0.95 ± 0.12 per 1,000 bases) in untreated mice to 0.12 ± 0.03 (or 0.17 ± 0.04 per 1,000 bases) after treatment with anti-DEC-OVA (Fig. 2f). Together, our results indicate that SHM rates per daughter cell decrease when GC B cells are forced, either genetically or pharmacologically, to undergo more inertial DZ cycles—once again arguing against a uniform rate of mutation per round of cell division.

To gain insight into the mechanisms that underlie the suppression of SHM in inertially cycling cells, we first quantified the appearance of mutations over time in the anti-DEC-OVA setting. Although DEC-205-sufficient B cells proliferated substantially and accumulated markedly in the DZ between 0 h and 48 h after anti-DEC-OVA treatment (Fig. 2g,h), SHM in this population ceased completely during this expansion period, after which it resumed at a near-normal rate (Fig. 2i,j and Extended Data Fig. 4b). Thus, the suppression of SHM after treatment with anti-DEC-OVA is not due to an even slowdown in mutation rates throughout the response, but rather to a temporary cessation of SHM during inertial cycling, followed by a return to the baseline rate at around the time of LZ re-entry.

**Fig. 3:**
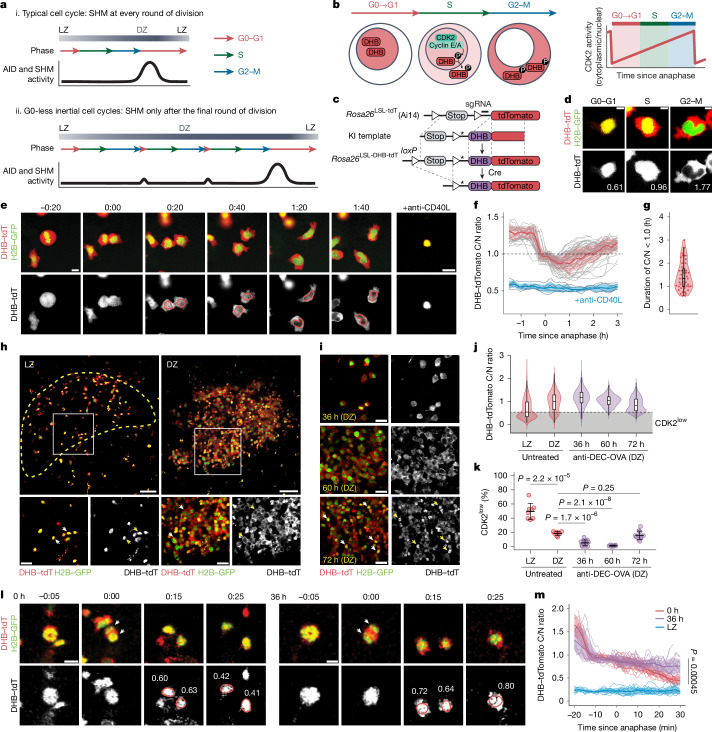
Inertial DZ cell cycles lack a CDK2^low^ G0-like phase. **a**, Proposed model for AID and/or SHM activity. **b**, Detecting CDK2 activity through subcellular translocation of a DHB reporter. **c**, *Rosa26*^DHB-tdTomato^ knock-in (KI) allele design. Asterisk denotes a silent mutation introduced to prevent sgRNA binding in the KI template. **d**, DHB–tdTomato (red) and H2B–GFP (green) in cultured GC B cells, with corresponding C/N ratios. Scale bars, 5 μm. **e**, Snapshots from time-lapse imaging of GC B cells in Nojima cultures expressing H2B–GFP (green) and DHB–tdTomato (red at the top, greyscale at the bottom; Supplementary Video 1). Scale bar, 10 µm. **f**, CDK2 activity traces (grey) with mean ± s.d. (red). Cells treated with anti-CD40L blocking antibody (blue) provide a CDK2^low^ reference. **g**, Duration of the C/N ratio below 1.0 (S-phase entry) after anaphase. Each symbol represents one daughter cell. **h**, DHB–tdTomato (red) and H2B–GFP (green) in GC B cells. CD35 expression (yellow dotted line) delineates the LZ (Supplementary Video 2). Arrowheads in insets highlight nuclear DHB–tdTomato in LZ cells and cytoplasmic expression in DZ cells. Scale bars, 50 µm (top); 20 µm (bottom). **i**, DHB–tdTomato (grey) after treatment with anti-DEC-OVA. Arrowheads highlight nuclear DHB–tdTomato at 72 h. Scale bars, 20 µm. **j**, Violin plot of CDK2 activity with box plot overlay (median, interquartile range, range). The grey box indicates the CDK2^low^ state (median of untreated LZ cells). Individual GC data are in Extended Data Fig. 6b,c. **k**, Fraction of CDK2^low^ cells in paired LZ and DZ cells in untreated GCs and DZ B cells after treatment with anti-DEC-OVA. *P* values by Student’s *t*-test. **l**, Snapshots of DHB–tdTomato (red) and H2B–GFP (green) from intravital time-lapse imaging at 0 h or 36 h after treatment with anti-DEC-OVA (Supplementary Videos 3 and 4). Images are aligned to the time of anaphase, determined by sister chromatid separation (arrowheads). CDK2 activity is indicated for each daughter cell. Scale bars, 10 µm. **m**, In vivo CDK2 activity traces in DZ cells with or without treatment with anti-DEC-OVA, summarized with mean and s.d. Non-dividing LZ cells (blue) serve as a CDK2^low^ reference. *P* value by Student’s t-test.

**Extended Data Fig. 5:**
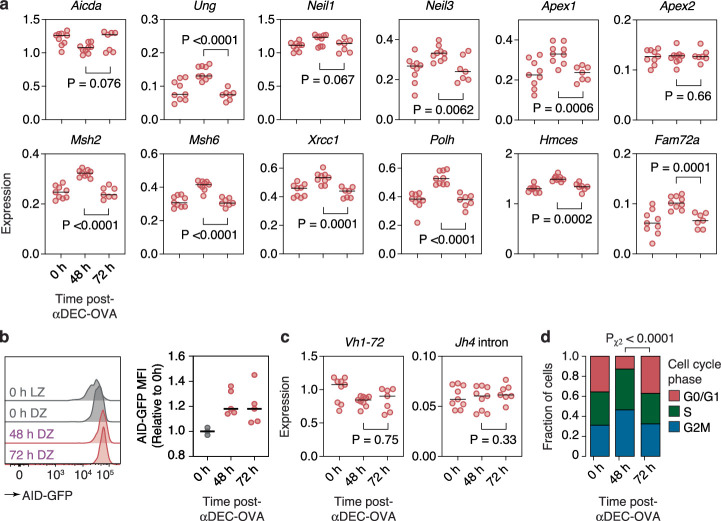
Gene expression in inertially cycling B cells. The anti-DEC-OVA-treated and control GC B cells analysed for mutational load in Fig. 2g–j were profiled for expression of genes involved in SHM. **a**, Expression of *Aicda* and downstream DNA repair genes in GC B cells prior to and at 48 and 72 h post-treatment. P values are given for the comparison between treated cells at 48 h post-anti-DEC-OVA (not actively undergoing SHM) and 72 h post-anti-DEC-OVA (actively mutating). Each symbol represents the mean expression value of single B cells from one mouse. Data are from two independent experiments. Expression of *Ung*, *Apex1*, *Apex2*, *Neil1*, *Neil3*, and *Xrcc4* (involved in base excision repair); *Msh2* and *Msh6* (required for mismatch repair); and the error-prone polymerase *Polh*, *Hmces*, and *Fam72a* (involved in the error-prone processing of AID-induced lesions) was not lower at 48 h. **b**, Expression of an AID-GFP fusion reporter analysed by flow cytometry at different times after anti-DEC-OVA treatment. *Left*, representative histograms; right, quantification of 2–4 samples for each time point. Each symbol represents one mouse. **c**, Detection of *Vh1-72* mRNA (encoding for the heavy chain of the B1-8^hi^ B cell receptor) and of the intronic RNA downstream of all Ighj segments (Jh4 intron, indicative of active Igh transcription) in GC B cells prior to and at 48 and 72 h post-anti-DEC-OVA treatment. Each symbol represents the mean expression value of single B cells from one mouse. Data are from two independent experiments. **d**, Gene-expression-based cell-cycle assignments for GC B cells from each time point (data as in **a**,**b**). Cells are pooled from 7–9 mice from 2 independent experiments as in **a**,**c**. Cell-cycle categories are assigned using the CellCycleScoring function in Seurat. G0/G1 cells are defined as those lacking expression of S-phase and G2/M-phase transcripts.

A comparison of B cell gene expression at 48 and 72 h after anti-DEC-OVA treatment ruled out downregulation of *Aicda* (which we confirmed by flow cytometry using an AID-GFP fusion reporter^31^) as the mechanism for this pause in SHM (Extended Data Fig. 5a,b). Moreover, expression of downstream effectors of SHM (*Ung*, *Neil1*, *Neil3*, *Apex1*, *Apex2*, *Msh2*, *Msh6*, *Polh*, *Hmces* and *Fam72a*; refs. ^32–38^) was generally higher rather than lower at 48 h after anti-DEC-OVA treatment, although fold changes were modest (Extended Data Fig. 5a). There was also no detectable downregulation of Igh mRNA or of Jh4 intronic RNA (a real-time indicator of *Ighv* transcription), suggesting that transcription of the *Ighv* region, a requirement for AID targeting^39^, is intact during inertial cycling (Extended Data Fig. 5c). By contrast, inertial cycling was associated with a sharp decrease in the proportion of B cells assigned transcriptionally to the G0–G1 phase of the cell cycle (Extended Data Fig. 5d). This is in line with our previously published findings in the *Ccnd3*^*T283A*^ mutant^21^ and indicates that B cells spend less time in the early phases of the cell cycle while undergoing inertial divisions. Given the established restriction of SHM to the earlier cell-cycle stages^40–42^, we hypothesized that B cell lineages undergoing clonal bursting in the DZ would be unable to undergo SHM except during the final post-mitotic phase that takes place between the end of inertial cell cycling and return to the LZ. Such a regimen would be equivalent to undergoing one round of SHM per DZ passage, rather than one per cell division (Fig. 3a). To test this hypothesis, we sought to measure the length of time B cells spend in G0–G1 under conditions of normal or clonal-burst-type cycling.

The G0 and G1 phases of the cell cycle are characterized by low activity of cyclin-dependent kinase 2 (CDK2), which, when partnered with cyclin E, promotes progression through G1 and S-phase initiation^43^. To measure these phases precisely in vivo, we generated mice carrying a real-time reporter of CDK2 activity (Fig. 3b,c). This reporter, based on previously published constructs^43,44^, consists of amino acids 994–1,087 of human DNA helicase B (DHB) fused N-terminally to the tdTomato red fluorescent protein and inserted into the ubiquitously expressed *Rosa26* locus (*Rosa26*^DHB-tdTomato^; Fig. 3c). The DHB domain translocates from nucleus to cytoplasm after phosphorylation by CDK2. It therefore localizes to the nucleus when CDK2 activity is absent during G0, gradually exiting to the cytoplasm as cells progress through G1, S and G2 phases^43,45^ (Fig. 3b,d). This localization-based readout for CDK2 activity, determined as the cytoplasmic-to-nuclear (C/N) ratio of DHB–tdTomato fluorescence (Fig. 3b), allows for monitoring of cell cycle progression with greater time resolution than can be achieved using conventional genetically encoded cell-cycle indicators^46^ that require transcription, translation and maturation of fluorescent proteins to take place before a signal can be detected. *Rosa26*^DHB-tdTomato^ mice were crossed to the B1-8^hi^ B cell receptor and a histone 2B (H2B)-EGFP reporter^47^ to accurately visualize mitotic events.

**Extended Data Fig. 6:**
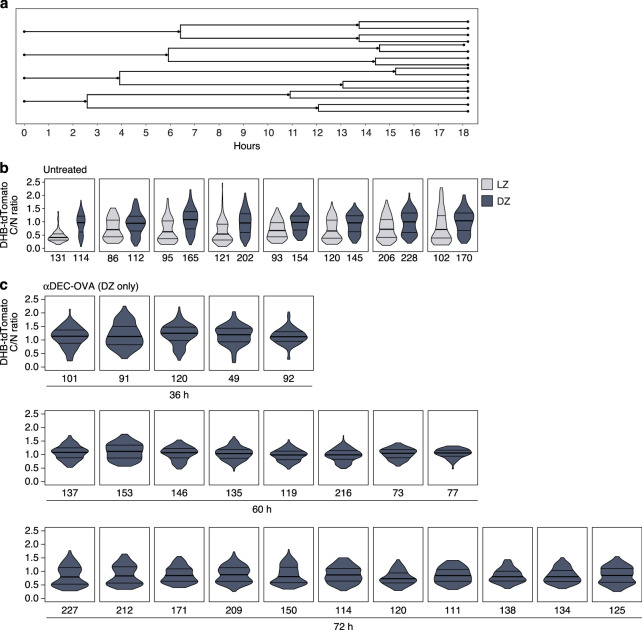
Measuring CDK2 activity in inertially cycling GC B cells. **a**, Lineage tracing of cells undergoing division in Nojima cultures. Each line represents one cell. Bifurcations represent cell divisions. **b**, Violin plots of DHB–tdTomato C/N ratio in LZ or DZ B cells within untreated GCs, segregated by individual GC and displayed with median and interquartile range. The number of cells quantified for each GC is indicated below each violin. Aggregated data are shown in Fig. 3j. **c**, Violin plots of DHB–tdTomato C/N ratio in DZ B cells within GCs 36, 60, or 72 h post-anti-DEC-OVA treatment, segregated by individual GC and displayed with median and interquartile range. Numbers of cells sequenced are indicated below each violin. Aggregated data are shown in Fig. 3j.

To test our reporter, we longitudinally imaged DHB–tdTomato^+^ GC B cells sorted onto feeder cell monolayers expressing CD40L, BAFF and IL-21 (‘Nojima’ cultures), which, in combination, are potent stimulators of GC B cell proliferation ex vivo^48,49^ (Supplementary Video 1). To follow the behaviour of our reporter during the transition from M phase to the following S phase, we aligned multiple single-cell DHB–tdTomato localization traces in time using anaphase as a reference (Extended Data Fig. 6a). We were unable to observe any cells entering a CDK2^low^ state, in which DHB–tdTomato is fully nuclear, as was the norm for GC B cells cultured in similar conditions but in the presence of a blocking antibody to CD40L (Fig. 3). Thus, when under constant stimulation, GC B cells never fully deactivated CDK2. Instead, immediately after mitosis, all cells exhibited an intermediate level of CDK2 activity, which continued to build and reached a C/N ratio of 1.0 (the reported threshold for the initiation of DNA synthesis^43^) within a median time of 1.3 h after anaphase, out of an average total cell-cycle duration of 8.7 h (Fig. 3g). Therefore, GC B cells under strong mitogenic stimulation are able to transition rapidly from M to S phases of the cell cycle by maintaining CDK2 activity close to the threshold required for S-phase entry, transiting rapidly through G1 and effectively avoiding a CDK2^low^ G0 stage^43^.

To verify whether such rapid M-to-S-phase transition was also seen during inertial cycling in vivo, we injected anti-DEC-205-OVA into mice with ongoing GCs seeded with a minor fraction of DHB–tdTomato-expressing B1-8^hi^ B cells, as in Fig. 2a, and imaged GCs both statically and dynamically using intravital imaging windows^50,51^. Static analysis of untreated GCs revealed that LZ B cells predominantly showed nuclear localization of DHB–tdTomato (median C/N ratio of 0.53; Fig. 3h,j,k, Extended Data Fig. 6b and Supplementary Video 2). Given that LZ B cells remain mainly in a quiescent G0-like state while competing for positive selection signals, we set the median of LZ B cells (0.53) as the threshold to define the CDK2^low^ state. In DZ B cells, by contrast, DHB–tdTomato was frequently enriched in the cytoplasm (median C/N ratio of 1.00 and 18.3% of the cells in the CDK2^low^ state; Fig. 3h,j,k, Extended Data Fig. 6b and Supplementary Video 2), indicative of active proliferation. Treatment with anti-DEC-205-OVA markedly increased the proportion of DZ B cells in active cell cycle (median C/N ratio of 1.2 and 1.1; 4.9% and 1.0% of cells in the CDK2^low^ state at 36 and 60 h after anti-DEC-OVA treatment, respectively), suggesting that inertially cycling DZ B cells mostly avoid a G0-like phase, instead maintaining an intermediate level of CDK2 activity after division (Fig. 3i–k, Extended Data Fig. 6c and Supplementary Video 2). By 72 h after treatment, when B cells are still predominantly in the DZ but anti-DEC-205-OVA-induced inertial proliferation is subsiding^21^, the median C/N ratio fell to 0.81, with 16% of DZ B cells in a CDK2^low^ state (Fig. 3i–k, Extended Data Fig. 6c and Supplementary Video 2).

Intravital time-lapse imaging of actively cycling DZ B cells showed that, at 36 h after anti-DEC-OVA treatment, C/N ratios in DZ B cells remained steadily above the CDK2^low^ G0 threshold (we were unable to track B cells for long enough to detect the increase in C/N ratios observed in vitro), whereas, in the absence of treatment, C/N ratios continued to decrease after mitosis, reaching the CDK2^low^ threshold within 30 min of anaphase (Fig. 3l,m and Supplementary Videos 3 and 4). Thus, B cells actively undergoing inertial cycling in response to anti-DEC-OVA largely failed to enter a CDK2^low^ G0-like state.

**Fig. 4:**
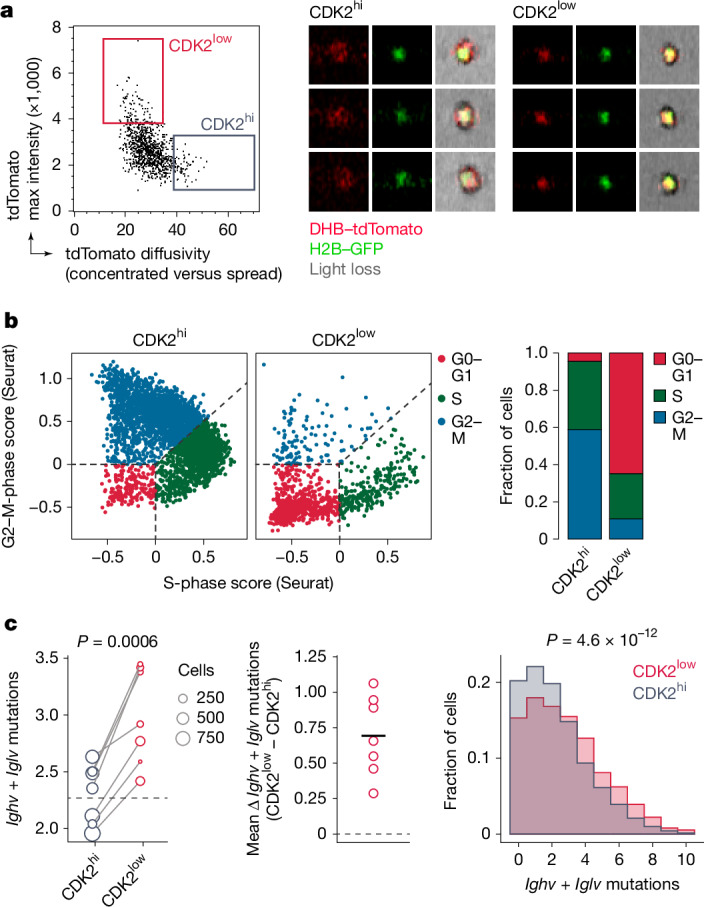
SHM takes place in DZ B cells in the CDK2^low^ G0-like state. **a**, Sorting gate (left) and examples of GC B cells with nuclear (CDK2^low^) and cytoplasmic (CDK2^hi^) DHB–tdTomato reporter as identified by image-based cell sorting (right). See Extended Data Fig. 7a,b for full sort strategy and further examples. **b**, Left, assignment of cell-cycle phase to CDK2^hi^ and CDK2^low^ DZ B cells by expression of S-phase and G2–M transcriptional signatures. Right, quantification. Cell-cycle categories are assigned using the CellCycleScoring function in Seurat. G0–G1 cells are defined as those lacking expression of S-phase and G2–M-phase transcripts. **c**, Left, number of *Ighv* + *Iglv* mutations in CDK2^low^ and CDK2^hi^ B cells 72 h after treatment with anti-DEC-OVA. Each symbol represents the mean of one mouse and is scaled according to the number of cells sequenced; line is the mean of all CDK2^low^ B cells. *P* value by paired *t*-test. Middle, difference between mean CDK2^hi^ and CDK2^low^ B cells in each mouse. Right, distribution of mutations in pooled CDK2^low^ and CDK2^hi^ B cells. Horizontal black line shows the mean. *P* value by Kolmogorov–Smirnov test.

**Extended Data Fig. 7:**
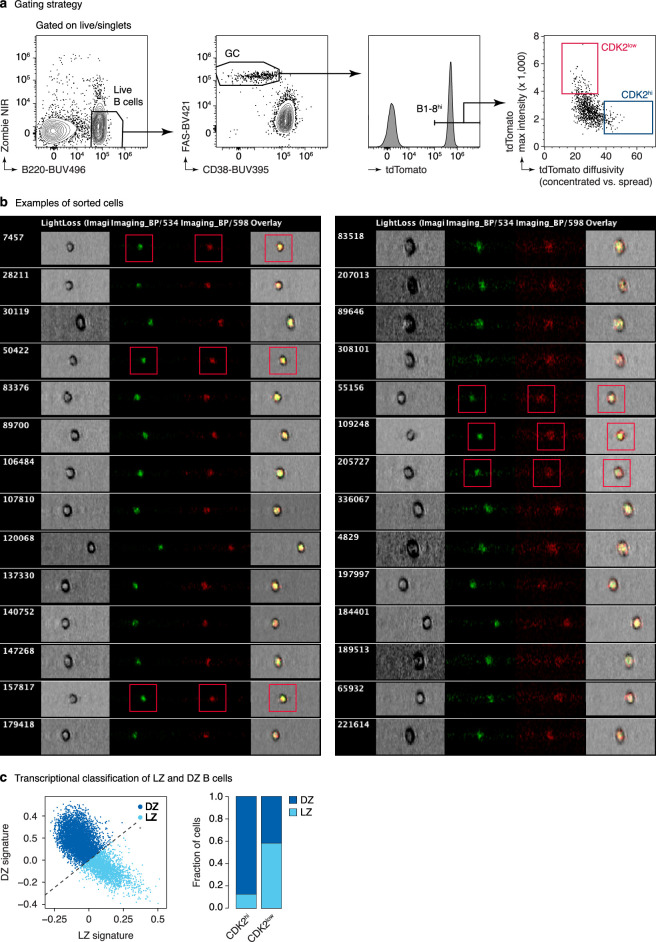
Image-based cell sorting of GC B cells by CDK2 reporter localization. **a**, Gating strategy used to sort cells with nuclear vs. cytoplasmic CDK2 reporter. Final panel is reproduced in Fig. 4a. **b**, Additional examples of images of sorted CDK2^hi^ and CDK2^low^ GC B cells. Red squares highlight the cropped images shown in Fig. 4a. **c**, Scatter plot used to classify cells as DZ or LZ using signatures described previously^68^.

To demonstrate the relationship between SHM and the CDK2^low^ state, we sought to measure the mutational load specifically among CDK2^low^ cells at 72 h after anti-DEC-OVA treatment. We used image-based cell sorting coupled with single-cell mRNA sequencing to isolate GC B cells with nuclear (CDK2^low^) versus cytoplasmic (CDK2^hi^) DHB–tdTomato reporter and a DZ transcriptional phenotype (Fig. 4a and Extended Data Fig. 7a–c). We found the expected strong association between nuclear DHB–tdTomato and a G0–G1 transcriptional state (that is, a lack of S- or G2–M-phase gene-expression programs) among DZ B cells (Fig. 4b). Crucially, as predicted by our model, CDK2^low^ DZ cells had on average 0.69 (±0.28, s.d.) additional mutations in *Ighv* + *Iglv*, compared with CDK2^hi^ DZ cells from the same sample (Fig. 4c), confirming that somatic mutation indeed takes place when B cells are in a CDK2^low^ state. Together, our data show that SHM is restricted to DZ B cells in the G0-like phase of the cell cycle, which indicates that the suppression of SHM that is evident during inertial cycling is, at least in part, attributable to the widespread skipping of this phase during inertial cell cycling.

Finally, to gain insight into how restricting SHM during inertial cycling might affect clonal selection and affinity maturation in GCs, we simulated full GC reactions in silico, whereby we could artificially turn off the ability of GC B cells to suppress SHM during inertial cycling. We adapted an established agent-based model of the GC reaction capable of realistic clonal bursting^7,9,26,52^ (see Methods and Supplementary Text) to include two scenarios: (i) a strict version of the model in Fig. 3a, in which B cells downregulate SHM to zero during inertial cycling and are completely barred from mutating until after the last round of DZ division before returning to the LZ (LAST); and (ii) a model in which this feature is disabled, and SHM is kept at a constant rate during each round of DZ division (EACH). When cells were allowed to mutate, the expected number of mutations in a descendant cell compared to its parent was set to 2.2, so as to yield an average SHM rate of 0.66 mutations per generation in LAST. We performed a total of 1,000 simulations for each scenario; GCs were simulated for 20 days after initial coalescence (corresponding to approximately 23 days in vivo), and late GCs were removed from analysis when they contracted to fewer than 200 cells.

**Fig. 5:**
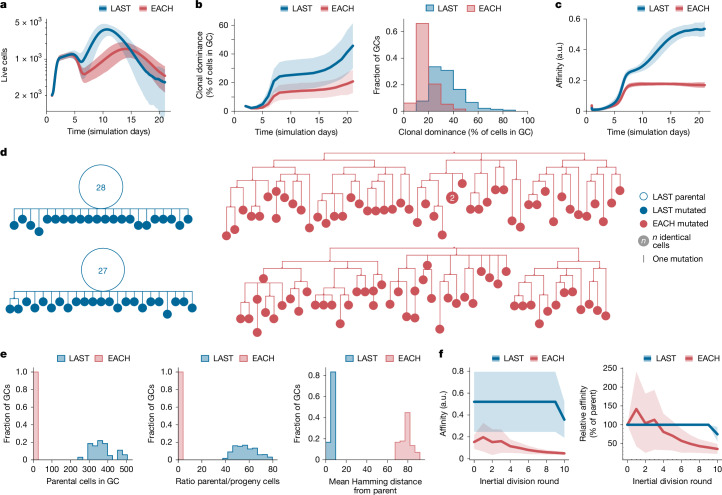
Dynamic regulation of SHM is required for simultaneous clonal expansion and affinity maturation in silico. **a**–**c**, One thousand in silico GC reactions were performed for each of two scenarios: EACH, in which B cells can undergo several divisions per DZ round, in a manner that depends on the amount of help they received in the LZ, and are free to mutate after every cell division; and LAST, which works as in EACH except that B cells can mutate only after the last inertial division of a DZ round. **a**, Number of live GC B cells. **b**, Percentage of B cells originating from the same founder B cell clone (clonal dominance) over time and frequency distribution of clonal dominance in GCs at day 14. **c**, Mean affinity of GC B cells in arbitrary units (a.u.) for the 1,000 simulations performed using each model. **d**, Example phylogenetic trees constructed from a sample of 100 cells drawn from day-14 phylogenies that had undergone ten consecutive inertial cell divisions. **e**, Quantification of data as in **d** across all bursting phylogenies. Distribution of bursting phylogenies according to the number of parental cells (left) and ratio of parental to progeny cells (middle) in each phylogeny. Right, mean mutational (Hamming) distance of all cells in the phylogeny to the cell that initiated the burst. **f**, Left, mean absolute affinity of GC B cells in a.u. Right, change in affinity in relation to the parental node for cells in the course of all bursts reaching ten divisions. Calculations in **e**,**f** are for the entire phylogeny rather than for the 100-cell samples shown in **d**. Shaded areas around the mean indicate s.d.

EACH GCs, in which the downmodulation of SHM during clonal bursting is disabled, were smaller in size and kinetically delayed compared with GCs generated by the LAST model (Fig. 5a). Both the rate of clonal expansion—measured as the percentage of each GC accounted for by its largest clone—and the average affinity of GC B cells were markedly higher in LAST GCs (Fig. 5b,c), suggesting that the expansion of high-affinity B cell clones in GCs fails in the presence of constant SHM. To investigate the effects of SHM downregulation at the phylogenetic level, we generated trees for all GCs containing clones with dominance greater than 75% in the LAST and EACH models. To mimic experimental conditions, samples of 100 cells were drawn from each GC, and all cells belonging to the bursting clone were included in the phylogeny. LAST phylogenies exhibited large parental nodes containing multiple cells with identical sequence, whereas EACH phylogenies were more branched and generally lacked expanded nodes (Fig. 5d), qualitatively matching the phenotypes of bursting and non-bursting GCs in our experimental data (Fig. 1b,c and Extended Data Fig. 1). Accordingly, the number of cells in the largest node of each bursting phylogeny was notably higher (193 ± 186 versus 0.69 ± 0.60 cells), and the mutational (Hamming) distance between the bursting cell and its progeny was much lower (0.57 ± 0.12 versus 7.7 ± 0.59 mutations) in LAST compared to EACH (Fig. 5e). To verify the prediction that sustaining SHM rates during inertial cycling would lead to a degradation of affinity in the progeny of a clonal burst, we selected phylogenies that underwent ten consecutive rounds of inertial division in both models, and plotted the average affinity of B cells after each round of division. As expected, clonal bursting in the EACH model led to an average decay in affinity as cells proliferated, whereas bursting lineages in the LAST model maintained higher affinities until the final round of DZ division (Fig. 5f,g). We conclude that removing the constraints on SHM during inertial cycling in silico leads to a marked loss of affinity among the descendants of a clonal burst and an overall failure of affinity maturation.

Somatic mutation and clonal expansion—two of the hallmarks of the GC reaction—are crucial for producing large quantities of high-affinity antibodies, but would pose problems for affinity maturation were they to occur simultaneously^11–13^. We propose that the immune system solves this potential conflict by restricting SHM to a specific stage of the cell cycle that is unavailable to rapidly dividing DZ B cells until the end of inertial proliferation, such that B cells undergo one round of mutation per DZ passage rather than per cell cycle (Fig. 3a). In this framework, clonal bursts closely resemble the behaviour of B cells in the original version of the cyclic re-entry model, undergoing several rounds of mutation-free division before each round of SHM^11^. However, because this model did not recapitulate the experimentally determined zonal distribution and migration kinetics of average GCs, it was subsequently replaced by its authors by a model in which B cells recycled more frequently between zones, with limited proliferation in each DZ passage^12^. Our findings suggest that these two versions of cyclic re-entry represent extremes of the same distribution of GC selection outcomes. At one end of the spectrum, weakly selected B cells undergo only a single round of division, followed by SHM, before returning to the LZ. This scenario converges with a model in which SHM takes place after every mitosis, and is roughly consistent with average GC kinetics^12^, given that, in steady state, GC B cells undergo an average of two divisions per DZ passage^10^. At the other extreme, clonal bursts represent exceptional deviations from average GC behaviour in which strongly selected B cell lineages expand several hundred-fold in a single DZ passage^11^. It is in this extreme scenario that the silencing of hypermutation during most division cycles becomes experimentally evident.

Mechanistically, the simplest explanation for an association between inertial cycling and dampened SHM would be that the total time a cell spends in the G0–G1 phase is shortened during inertial expansion, allowing less time for AID to act on Ig genes before the onset of DNA replication. Such a model is in agreement with the relatively slow rate of AID deamination of cytosines in vitro^53^. However, our findings suggest that the key difference between steady-state and inertially cycling GC B cells is that the latter specifically fail to access a state of low or no CDK2 activity. Emerging views on the regulation of cell-cycle entry indicate that, in continuously cycling cells, low CDK2 activity characterizes a temporary quiescent phase of the cell cycle—G0 or at least ‘G0-like’—which can be skipped entirely in cells that are proliferating very rapidly^43^. This raises the possibility that specific features of the DZ G0-like state either enhance AID activity or favour error-prone over faithful repair of the uracils it creates in DNA^32–35^. For example, it was recently shown that D-type cyclins, which are highly expressed during DZ inertial cycles^21^, inhibit the recruitment to DNA lesions of components of the error-prone mismatch repair (MMR) pathway^54,55^, instead favouring more faithful base excision repair (BER) in G0–G1 (ref. ^56^). Thus, a potential mechanism for the downregulation of SHM during inertial cycles is that high cyclin D activity keeps error-prone MMR at bay. This possibility is in line with our finding that mice in which cyclin D3 is stabilized show decreased SHM rates per cell division. However, it would require an additional mechanism operating during inertial cycling to ensure that AID-catalysed uracils are repaired faithfully by BER, which in GC B cells can also be mutagenic^35^.

A dynamic rate of SHM in GC B cells had been invoked in previous mathematical models precisely because, in silico, reducing mutational probabilities in high-affinity cells, which are those that divide the most, was found to improve affinity maturation^9,57^. The mechanism proposed here—namely, that mutation is limited to the last division of each DZ round—aligns with that prediction by reducing the number of mutations per DZ passage specifically in high-affinity cells, which are those most likely to undergo inertial proliferation in the DZ. Nonetheless, completely eliminating SHM during inertial cycles yields phylogenies that are more strongly dominated by the parental node than are those we observed in vivo (compare Fig. 1b and Extended Data Fig. 1c to Fig. 5d). Barring technical explanations related to imprecisions in the timing of clonal bursts in relation to sampling in vivo, this suggests that mutation is strongly suppressed but may not be entirely absent during inertial cycling.

In conclusion, our data support a model in which B cells silence SHM during inertial cycling, so as to undergo one (or possibly a few) SHM cycles per DZ passage, as opposed to one round per cell cycle. This model reconciles two salient features of GC biology—cyclic re-entry and clonal bursting—and explains how GCs can simultaneously achieve the efficient clonal expansion and sequence diversification that are required for antibodies to affinity mature.

## Methods

### Mice

C57BL/6, B6.SJL (CD45.1), *Rosa26*^Confetti^ (ref. ^24^) and H2B-EGFP-transgenic^47^ mice were purchased from Jackson Laboratories (strains 000664, 002014, 017492 and 006069, respectively). *Aicda*^CreERT2^ mice^23^ were provided by C.-A. Reynaud and J.-C. Weill. *Col1a1*^tetO-H2B-mCherry^.Vav-tTA mice^10,58,59^ were provided by M. Nussenzweig. *Cd79a*^Cre^ mice^60^ (Jax strain 020505) were provided by M. Reth. *Ccnd3*^T2873A^ mice were generated in our laboratory as previously described^21^. *Igh*B1-8^hi^ (ref. ^61^) and *Ly75*^−/−^(ref. ^62^) mice were bred and maintained in our laboratory. DHB-tdTomato mice were generated in our laboratory by CRISPR–Cas9-mediated insertion of amino acids 994–1,087 of human DNA helicase B (DHB)^43,44^ upstream of the start codon of tdTomato of *Rosa26*^loxP-stop-loxp-tdTomato^ (Ai14) mice^63^ (Jax strain 007914) so as to create an N-terminal fusion protein. The targeting sgRNA (5′-CAAGCTAGATCGAATTCGGC-3′) was purchased from Integrated DNA Technologies. The insert encoding for the DHB fragment flanked by 400-bp homology arms was delivered by adeno-associated virus (AAV) into fertilized mouse oocytes using the CRISPR-READI protocol as described^64^. Correct insertion of the allele was verified by Sanger sequencing across the entire locus using genomic primers positioned outside the homology arms. To minimize potential CRISPR off-target effects, the founder mouse was back-crossed for at least five generations onto C57BL/6J mice before experimental use. Mice were bred and maintained under specific-pathogen-free conditions at the Rockefeller University Comparative Biology Center. All animal procedures were approved by the Institutional Animal Care and Use Committee of the Rockefeller University. All experiments were performed using male and female mice aged 5–12 weeks.

### AID-Confetti labelling

*Rosa26*^Confetti/Confetti^*.Aicda*^CreERT2/+^ mice were immunized subcutaneously in the footpad with 10 µg chicken IgY (Exalpha Biologicals) in Imject Alum (Thermo Fisher Scientific) at a 2:1 v:v ratio. *Rosa26*^Confetti^ recombination was induced by either one gavage at 5 dpi or two gavages at 5 dpi and 7 dpi (see Supplementary Data 1), of 10 mg of tamoxifen (Sigma, T5648) dissolved in corn oil at 50 mg ml^−1^.

### Multiphoton imaging and analysis of Brainbow GCs

For Brainbow imaging, lymph nodes (LNs) were collected at the indicated times after immunization and mounted in phosphate-buffered saline (PBS) between two coverslips held together using vacuum grease, as described previously^7^. Mounted pLNs were imaged on an Olympus FV1000 upright microscope equipped with an Olympus 25× 1.05 NA Plan water-immersion objective and a Spectraphysics Mai-Tai DeepSee Ti-Sapphire laser tuned to an excitation of λ = 930 nm. Emission was detected using a pair of CFP (480/40 nm) and YFP (525/50 nm) filters, separated by a 505-nm dichroic mirror for CFP, GFP and YFP detection and a separate RFP filter (605/70 nm). The NDS for each GC was determined by multiplying the fraction of fluorescent cells (determined by the density of fluorescent cells in the DZ) by the fraction of cells of the most frequent Confetti colour, as described previously^7^.

### Isolation of single GCs for flow cytometry and cell sorting

Individual AID-Confetti GCs were isolated as described previously^7^ by embedding pLNs in 4% low-melt NuSieve GTG agarose in PBS. LNs were cut into 300-µm slices on a Leica VT1000A vibratome. Slices were then further dissected into single-GC fragments using a double-edged razor blade under a Leica M165FC fluorescence stereomicroscope. Slice fragments were macerated using micropestles in microcentrifuge tubes containing 100 µl PBS supplemented with 0.5% bovine serum albumin (BSA) and 2 mM EDTA (PBE), followed by gentle vortexing. Suspensions were stained by adding 100 µl of a 2× antibody cocktail (CD38, FAS, B220, TCRαβ and Fc block; see Extended Data Table 1) and were single-cell index-sorted on a BD FACSymphony S6 sorter. Colour assignment was done after acquisition using Diva software (v. 8.0.2).

### Adoptive cell transfers

Splenocyte suspensions were obtained by forcing spleens from B1-8^hi^ tTA-H2B-mCherry mice (*Igh*^B1-8hi/+^.*Col1a1*^tetO-H2B-mCherry/WT^.Vav-tTA-transgenic CD45.2/2) through a 40-µm strainer, then lysing red blood cells with ACK buffer (Lonza), before resuspension in PBE. Resting B cells were isolated by negative selection using anti-CD43 magnetic beads (MACS, Miltenyi Biotech), as per the manufacturer’s instructions. Before transfer, the percentage of NP-binding B1-8^hi^ cells was determined by flow cytometry of splenocytes stained with 5 µg ml^−1^ of NP(19)-phycoerythrin (APC) (conjugated in-house). A total of 5 × 10^5^ NP-binding B cells was transferred into each recipient. To induce GCs, recipient CD45.1/1 mice were primed intraperitoneally with 50 µg OVA in Imject Alum (Thermo Fisher Scientific) at a 2:1 v:v ratio in a final volume of 100 µl, two to four weeks before cell transfer. Purified B1-8^hi^ B cells were adoptively transferred at the proportion indicated in each figure. The following day, mice were immunized subcutaneously in the footpad with 25 µg NP(19)-OVA (Biosearch Technologies) in Imject Alum as above. Seven days after immunization, mice were treated with 5 µg of anti-DEC-OVA, produced in our laboratory as described^65^, in PBS, injected subcutaneously into the footpad. For division tracking, mice were given 1.6 mg DOX (Sigma) by intraperitoneal injection 12 h after treatment with anti-DEC-OVA, and maintained on DOX throughout the remainder of the study by adding DOX (2 mg ml^−1^) and sucrose (10 mg ml^−1^) to the drinking water. All treatment groups were randomly assigned.

### Plate-based single-cell VDJ sequencing and analysis

For plate-based single-cell Ig sequencing (Fig. 1 and Extended Data Figs. 1 and 3c–e), GC B cells were index-sorted as above into 96-well plates containing 5 μl TCL buffer (Qiagen) and 1% β-mercaptoethanol. RNA from each cell was extracted and reverse-transcribed using oligo-dT primers, and the Ig heavy chains were amplified by PCR, as previously described^7^. The PCR products were combined, purified using SPRI beads and sequenced with the 500-cycle Reagent Nano kit v2 for single-cell libraries using the Illumina platform. All sequences are provided in Supplementary Data 2.

For single-cell *Ighv* analyses, raw paired-end sequences were merged using PANDAseq (v.2.11) for full amplicon reconstruction^66^ and then processed with the FASTX toolkit. Only Ig sequences with high counts for each single cell were filtered for further analysis and were submitted to the HighV-QUEST (v.1.6.9) database^67^ for V-(D)-J gene rearrangement annotation. Sequences that shared V_H_/J_H_ genes with identical CDR_H_3 lengths were classified into clonal lineages if the CDR_H_3 nucleotide identity was at least 75%. Clonal lineage trees were constructed using gctree^27^, and are rooted on the unmutated germline V-gene sequence of that particular clone. GC B cells were included in lineages regardless of their colour, which led to the inclusion of a small number of cells that were not of the dominant colour of the burst. These are likely to represent cells that changed colour after the burst was induced, owing to residual tamoxifen activity. For each clone, the top-ranked reconstruction generated by gctree was always chosen. In phylogenies from single-coloured GCs, parental status was assigned post hoc to the node that contained the largest number of identical sequences, with the assumption that that node represents the B cell that gave rise to the clonal burst.

Clonal-burst sizes were extrapolated to a 2,000-cell GC as (number of cells at or below burst point) × (fraction of fluorescent cells) × [2,000/(total cells sequenced)], the fraction of fluorescent cells being determined from imaging-based cell density data as described above.

### Droplet-based single-cell VDJ and gene-expression sequencing and analysis

To generate 10X Genomics Ig and gene-expression libraries (Figs. 2d–j and 4c and Extended Data Fig. 4a,b), GC B cells were co-stained with individual hashtag oligonucleotide (HTO)-labelled antibodies to CD45 and MHC-I (Biolegend) for sample-level barcoding before sorting. Cells were pooled in a microfuge tube in PBS supplemented with 0.4% BSA and were counted for viability by trypan blue staining. Single-cell gene expression and B cell receptor (BCR) libraries were generated using the Chromium Next GEM Single Cell 5’ Reagent Kit v2 or v3(Dual Index) with Feature Barcode technology for Cell Surface Protein, according to the manufacturer’s protocol. Libraries were sequenced on a Nextseq2000 (Illumina) or an Aviti 500M (Element) flowcell with a minimum sequencing depth of 30,000 reads per cell. CellRanger v.8.0.1 was used alongside the mm39 mouse reference to generate unique molecular identifier (UMI) and HTO count matrices. BCR libraries were processed with CellRanger ‘vdj’ with default parameters. Transcriptomic analysis was performed using Seurat v.5.1.0. Cell-cycle categories were assigned using the CellCycleScoring function. LZ and DZ signatures^68^ were assigned using the AddModuleScore function. Only B1-8^hi^ cells with *Ighv1-72*/*Ighj2*–*Iglv1*/*Iglj1* pairing were filtered for further analysis.

### Time-lapse imaging of GC B cells in culture

NB-21.2D9 feeder cells expressing CD40L, BAFF and IL-21 (provided by G. Kelsoe) were maintained in DMEM supplemented with 10% heat-inactivated FBS and penicillin streptomycin solution (Corning). The cells were detached with trypsin and resuspended in OptiMEM, irradiated (20 Gy) and seeded into 96-well glass-bottom plates (Cellvis) at 3,000 cells per well in OptiMEM supplemented with 10% heat-inactivated FBS, 2 mM l-glutamine, 1 mM sodium pyruvate, 50 μM 2-ME, penicillin streptomycin solution, 10 mM HEPES, MEM vitamin solution (Sigma) and MEM non-essential amino acids (Gibco). The following day, 2,000 DHB–tdTomato- and H2B–GFP-expressing GC B cells were sorted into each well and supplemented with 150 μl of OptiMEM along with 30 μg ml^−1^ LPS (Sigma-Aldrich) and 4 ng ml^−1^ IL-4 (Thermo Fisher Scientific). Anti-CD40L blocking antibody (25 μg ml^−1^, Bio X Cell) was added as indicated. Time-lapse imaging was performed on CellDiscoverer7 (Zeiss) with a 20× objective. Cells were kept in a humidified, 37 °C incubator at 5% CO^2^. Images were taken in GFP and RFP channels every 5 min.

### Intravital imaging

Intravital imaging was performed using inguinal lymph node (iLN) windows as previously described^50,51,69^. In brief: on the day of imaging, custom-designed titanium windows were surgically mounted over the iLN under anaesthesia with 1.25% isoflurane in oxygen. Mice were placed on a specially designed heated stage with a fixture for window positioning under an Olympus FV1000 upright microscope as above. Four-dimensional (4D) datasets were acquired as 20 *z*-slices 5 µm apart (total 100 µm volume) with 2.0× zoom and 512 × 512 *x*–*y* resolution. Full *z*-stacks were acquired every one minute.

### Quantification of CDK2 activity

The nuclear mask was determined using the H2B–GFP channel, and the cytoplasmic mask was drawn as a band 0.5 µm outside the nuclear mask. Mean pixel intensities were measured for the nuclear and cytoplasmic masks in the DHB–tdTomato channel to calculate CDK2 activity as the cytoplasmic-to-nuclear intensity ratio. Considering the irregular shape and limited cytoplasmic area of GC B cells, the cytoplasmic band might overlap with extracellular space. To minimize this issue, we calculated a moving average of each cell’s CDK2 activity across three frames (the preceding, current and the following frames) and plotted this data in Fig. 3f,m.

### Image-based cell sorting

*Rosa26*^DHB-tdTomato^ or *Rosa26*^DHB-tdTomato^.H2B-GFP GC B cells from anti-DEC-OVA-treated mice (72 h after treatment) were collected as described above. Cell suspensions were stained with an antibody cocktail (B220, CD38, FAS, HTO and Fc block) and Zombie NIR (Biolegend) viability dye, then sorted on a BD S8 FACSDiscover spectral sorter with BDCellView Image Technology. Cells were sorted on the basis of the maximum intensity and diffusivity of tdTomato fluorescence, following the gating strategy indicated in Extended Data Fig. 7a,b.

### Simulation of clonal-burst birth–death processes

We used the Python package BDMS^28^ to generate phylogenies with a constant mutation probability of one-third for each daughter after a cell division. In this simulation, we assume a fixed unit birth rate (defining an arbitrary timescale). For each Brainbow GC sequenced, we simulate until the number of cells matches the empirical estimate from Brainbow bursts. Our simulation defines three types of cells: the parental unmutated type at the root of the burst; a mutant type; and a nonsense type, representing mutated cells that have acquired a stop codon. Daughters of parental cells mutate with a probability of one-third, and each mutant is nonsense with probability 3/63 (that is, on average 3 of the 63 codons to which a B cell can mutate are stop codons). Nonsense types immediately die. Code and results for these simulations are available at https://github.com/dewitt-lab/aid-sim.

### Estimation of GC B cell division and mutation rates

In Extended Data Fig. 3c, we sought to predict the expected number of accumulated mutations given different GC B cell proliferation histories. We first estimated the division rate in steady-state GCs from our previously published^21^ fraction of B cells entering S phase over a 30-minute period, measured using dual nucleotide pulsing^10^. Let *x* be the fraction of the DZ that enters S phase within a 30-minute window. On average, this means that the division period is *T* = 100/(2*x*), where *T* is measured in hours. More importantly, we can use this division period to estimate the expected number of mutations *m* after *t* hours, because we should expect only *t*/*T* divisions to occur. To be specific, we should predict the average number of mutations to be

$$m=\frac{\rho t}{T}=\frac{2\rho tx}{100},$$

where *ρ* is the per-division accumulation mutation rate. For the purpose of this figure, we take this to be 0.372, given the average *Ighv* length of 372 bases, and the null hypothesis of one mutation per 1,000 bases. Because of measurement error in both the time elapsed *t* and the division per cent *x*, we need to be careful to track error propagation. Using standard methods, we estimate the error in the mutation count to be

$${\sigma }_{m}=m\sqrt{{\left(\frac{{\sigma }_{t}}{t}\right)}^{2}+{\left(\frac{{\sigma }_{x}}{x}\right)}^{2}},$$

where *σ*_*t*_ is the error in the time elapsed and *σ*_*x*_ is the error in the 30-minute division per cent.

In Extended Data Fig. 3f, we calculated the per-daughter *Ighv* mutation rate using similar logic. Because we have the number of mutations *m* in the data, we can rearrange the previous equation to estimate the per-division mutation accumulation rate *ρ* via

$$\rho =\frac{100m}{2xt}.$$

Again, using standard error propagation, we get the error in the mutation rate to be

$${\sigma }_{\rho }=\rho \sqrt{{\left(\frac{{\sigma }_{t}}{t}\right)}^{2}+{\left(\frac{{\sigma }_{x}}{x}\right)}^{2}+{\left(\frac{{\sigma }_{m}}{m}\right)}^{2}},$$

where *σ*_*m*_ is naturally the error in the mean mutation count. If one wants the per-base-pair mutation rate, one needs to merely take *ρ*/372, because that is the length of the gene.

In Fig. 2d, predicted mutation counts at day 10 for both populations were estimated by taking the mutation count from a reference population measured at day 7, then using mCherry dilution data to estimate the number of divisions across this time. Taking these together, the equation becomes

$${m}_{10}={m}_{7}+\rho \eta d\tau .$$

Here, *m*_10_ is the average number of mutations at day ten, *m*_7_ is the average number of mutations at day 7 and *d* is the number of divisions that took place across those two days. *τ* is a time factor that was set at 3/2.5 for this experiment to reconcile the difference in timing between the observable mCherry dilution period (2.5 days since initiation of DOX treatment) and the anti-DEC-OVA-induced cell proliferation (3 days since injection of anti-DEC-OVA). *ρ* is the per-division mutation accumulation rate, which using the 1 in 1,000 bases null hypothesis sets *ρ* = 0.7, given the length of the *Ighv* and *Iglv* chains combined. *η* is the mutant survival rate, which is a subtle point about this calculation. Previously, the division rate (and therefore, total number of divisions) was determined by directly measuring the fraction of cells entering S phase. However, mCherry is only present in surviving cell lineages; it therefore paints an overly optimistic picture of division rates, because, in the real-life GC, deleterious mutations are presumed to be eliminated under selection. We assumed *η* = 0.5 for the day-10 population to account for selection in the untreated setting^6^. However, for the day-10 anti-DEC-OVA-treated population, selection is effectively turned off (because proliferation happens in the DZ in the absence of affinity-based selection), so *η* = 1.0. The error equation in this case is given by

$${\sigma }_{{{m}}_{10}}=\sqrt{{({\sigma }_{{{m}}_{7}})}^{2}+{(\rho \eta \tau {\sigma }_{{{d}}_{10}})}^{2}}.$$

Here, ${\sigma }_{{{m}}_{7}}$ is the uncertainty of the day-7 average mutational count and ${\sigma }_{{{d}}_{10}}$ is the uncertainty of the day-10 division number (although division number uncertainty is experimentally inconvenient to measure, it has been included here for didactic purposes).

In Fig. 2f, the per-division mutation accumulation rate was estimated by comparing the number of mutations at day 10 to the mutation count of a reference population at day seven, and then using mCherry dilution data to estimate the number of divisions across this time. Basically, we rearrange the previous equation to get

$$\rho =\frac{{m}_{10}-{m}_{7}}{\tau \eta {d}_{10}}.$$

The mutation rate is simply the difference in the number of mutations, divided by the number of divisions, multiplied by a factor correcting for the mutants that got picked off in selection. The error formula takes on the form

$${\sigma }_{\rho }=\left(\frac{1}{\tau \eta }\right)\sqrt{{\left(\frac{{\sigma }_{{{m}}_{10}}}{{d}_{10}}\right)}^{2}+{\left(\frac{{\sigma }_{{{m}}_{7}}}{{d}_{10}}\right)}^{2}+{\left(\tau \eta \rho \frac{{\sigma }_{{{d}}_{10}}}{{d}_{10}}\right)}^{2}}.$$

### Agent-based GC simulation model

We adapted a previously published agent-based simulation of the GC reaction^26,52^ to analyse the effects of different modalities of SHM regulation on GC clonal composition and affinity maturation. A full description of the model is provided in the Supplementary Text. In brief, the simulations start with a pool of founder B cells, which, after an initial round of proliferation, collect antigen from follicular dendritic cells and signals from T_FH_ cells in the LZ. Depending on the amount of signal provided by T_FH_ cells, B cells undergo successive rounds of proliferation in the DZ. The affinity landscape is represented in a 4D space (Shape Space). Mutation induces a randomly directed change of position with a distance of one from the previous position. The B cell mutation occurs after cell division, according to the mutation models described below.

After selection, each B cell undergoes a number of divisions, *n*, determined by the level of MYC at the time of selection. We implemented two models of B cell mutation during cell division.

- EACH scenario: every daughter cell acquires *M* mutations each division.
- LAST scenario: only daughter cells in the last round of proliferation acquire *M* mutations.

For both scenarios, *M* is set to 2.2; that is, each descendant undergoes two mutations relative to its parent and has a 0.2 probability of acquiring a third mutation. *M* was chosen so that the average mutation rate matches the observed value of 2/3 mutations per daughter cell in the LAST model.

In silico, DZ B cells naturally undergo an average of two divisions and, in extreme cases, ten or more divisions. For further details, see the Supplementary Text.

To analyse the mutational landscape of the GC, we translated the shape space positions into BCR sequences. The mutations of cells in the shape space are tracked throughout the course of the GC reaction. Each seeder cell is assigned a unique random nucleotide sequence of 300 base pairs, representing the *Ighv* segment of a real BCR sequence. Because we restricted the modelled sequence to the *Ighv* segment, mutations in the shape space correspond to a random nucleotide replacement in the *Ighv* sequence with a 50% reduced mutation frequency only.

Using our previously established in silico implementation of the ‘Brainbow’ model^7,26^, we selected, from a pool of 1,000 simulated GCs, those dominated by a single colour (more than 50%) (see Supplementary Text). We restricted the burst analysis (Fig. 5d–f) to clonal bursts of ten consecutive rounds of proliferation from the selected subset of GCs, and sequenced cells 70 h after the start of the burst.

### Statistical analysis

Statistical tests used to compare conditions, indicated in figure legends, were performed in R (v.4.3.1) or Python. No statistical methods were used to determine sample size. Flow-cytometry analysis was performed using FlowJo (v.10). The 95% CIs around the proportion of parental cells in a clonal burst were calculated online using the binomial exact method at https://sample-size.net/confidence-interval-proportion/. Graphs were plotted using R or Python and formatted in Adobe Illustrator CS.

### Reporting summary

Further information on research design is available in the Nature Portfolio Reporting Summary linked to this article.

## Online content

Any methods, additional references, Nature Portfolio reporting summaries, source data, extended data, supplementary information, acknowledgements, peer review information; details of author contributions and competing interests; and statements of data and code availability are available at 10.1038/s41586-025-08687-8.

## Supplementary information

Supplementary Text Detailed description of the agent-based simulation of the GC reaction used in Fig. 5.

Reporting Summary

Supplementary Data 1 Brainbow and lineage parameters for individual GCs (shown in **Fig. 1c** and **Extended Data Fig. 1b)**, used in simulations of clonal-burst birth–death processes presented in **Fig. 1c-f** and **Extended Data Fig. 2**.

Supplementary Data 2 Plate-based Ig sequencing results for **Fig. 1** and **Extended Data Figs. 1** and 3c–e.

Supplementary Video 1 Time-lapse imaging of H2B–GFP and DHB–tdTomato expressing GC B cells in Nojima cultures.

Supplementary Video 2 DHB–tdTomato and H2B–GFP expression in LZ or DZ B cells in GCs, treated or untreated with anti-DEC-OVA, in explant LNs.

Supplementary Video 3 Intravital time-lapse imaging of H2B–GFP and DHB–tdTomato expressing GC B cells.

Supplementary Video 4 Intravital time-lapse imaging of H2B–GFP and DHB–tdTomato expressing GC B cells 36 h post-anti-DEC-OVA treatment.

## Figures and Tables

**Extended Data Table 1:**
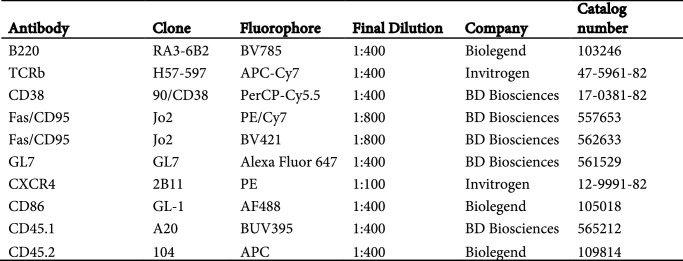
Antibodies used for flow cytometry

## Data Availability

Single-cell RNA-sequencing data and Seurat objects are available via the Gene Expression Omnibus under accession number GSE285185. Code and results for simulations branch-process simulations are available at https://github.com/dewitt-lab/aid-sim. All other data needed to evaluate the conclusions in this manuscript are available via Zenodo at 10.5281/zenodo.14516289 (ref. ^70^).
