# Supplementary material for: Transient silencing of hypermutation preserves B cell affinity during clonal bursting
Source: Nature. 2025 Mar 19;641(8062):486–94. doi: 10.1038/s41586-025-08687-8 (PMC12058519; doi:10.1038/s41586-025-08687-8)
Supplement: Supplementary file 1 — Detailed description of the agent-based simulation of the GC reaction used in Fig. 5. [file 41586_2025_8687_MOESM1_ESM.pdf]

nature portfolio

https://doi.org/10.1038/s41586-025-08687-8

Supplementary information

Transient silencing of hypermutation preserves B cell affinity during clonal bursting

In the format provided by the authors and unedited

Supplementary Material

November 13, 2024

1 Germinal Centre Simulation Model

The germinal center (GC) model assumptions underlying the GC simulations are explained here and include the used parameter values. It follows [1] with additional features [2, 3, 4], and adding the specificities of the DisseD model from [5]. Used acronyms are: DZ for dark zone, LZ for light zone, Tfh for T follicular helper cell, FDC for follicular dendritic cell, BC for B cell, BCR for B cell receptor.

1.0.1 Space representation

All reactions take place on a three-dimensional discretized space with a rectangular lattice with lattice constant of $\Delta x = 5\mu m$. Every lattice node can be occupied by a single cell only.

1.0.2 Shape space for antibodies

Antibodies are represented on a four ($d = 4$) dimensional shape space [6]. The shape space is restricted to a size of 10 positions per dimension, thus, only considering antibodies with a minimum affinity to the antigen. The optimal clone Φ^* is positioned in the center of the shape space. A position on the shape space Φ is attributed to each BC.

The 1-Norm with respect to the optimal clone $||\Phi - \Phi^*||_1 = \sum_{i=1}^{d} |\Phi_i - \Phi_i^*|$, i.e. the minimum number of mutations required to reach the optimal clone, is used as a measure for the antigen binding probability. The binding probability is calculated from the Gaussian distribution with width $\Gamma = 2.8$ [7]:

$$b\left(\Phi, \Phi^*\right) \quad = \quad \exp\left(-\frac{||\Phi - \Phi^*||_1^2}{\Gamma^2}\right) \quad . \tag{1}$$

1.0.3 B cell phenotypes

Three BC phenotypes are distinguished: DZ BCs, LZ BCs, and output cells. The different phenotypes characterize the cell properties and are not meant as localization within the GC zones. DZ BCs divide, mutate and migrate. LZ BCs also migrate and undergo the different stages of the selection process. Output cells only migrate.

1.0.4 Founder cells

The model starts with 150 Tfh, 200 FDCs, 300 stromal cells, and no BC. Tfh are randomly distributed on the lattice and occupy a single node each. Stromal cells are restricted to the DZ (see section *Chemokine distribution* for their function). FDCs are restricted to the upper half of the reaction sphere, occupy one node by their soma and have 6 dendrites of $40\mu m$ length each. The presence of dendrites is represented as a lattice-node property and, thus, visible to BCs. The dendrites are treated as transparent for BC or Tfh migration such that they do not inhibit cell motility.

BCs enter the GC reaction with a probability per time step corresponding to a rate of 2 cells per hour (for an estimation of this value refer to [4]). New BCs are randomly positioned on free lattice nodes. The shape space position of each new BC is randomly chosen but restricted to positions with a distance of at least 3 from the optimal clone.

1.0.5 Antigen-presentation by FDCs

Each FDC is loaded with 10000 antigen portions distributed onto the lattice-nodes occupied by FDC-soma or FDC-dendrite unless stated otherwise. One antigen portion corresponds to the number of antigen molecules taken up by a BC upon successful contact with an FDC.

1.0.6 DZ B cell division

The average cell cycle duration of 7.5 hours of DZ BCs is varied for each BC according to a Gaussian distribution. This is needed to get desynchronization of BC division. The cell cycle is decomposed into four phases (G1, S, G2, M) in order to localize mitotic events if this is needed.

Each founder BC divides a number of times before differentiating to the LZ phenotype for the first time. Six divisions was the number of divisions found in response to the extreme stimulus with anti-DEC205-OVA [8, 1]. Each selected BC divides a number of times determined by the interaction with Tfh (see below, LZ B cell selection). The parameters of the interaction with Tfh are tuned such that the mean number of divisions is in the range of two [9]. This value is required in order to maintain a DZ to LZ ratio in the range of two [8, 1].

A division requires free space on one of the Moore neighbors of the dividing cell. Otherwise the division is postponed until a free Moore neighbor is available.

Timing of B cell receptor mutation during internal cycling is model dependent. In the EACH model, cells mutate in each division round of internal cycling. Mutation in the LAST model is restricted to the last round of internal cycling. In both models is the number of acquired mutations N set to 2.2 for each mutation event i.e. each descendant undergoes two mutations relative to its parent and has a 0.2 probability of acquiring a third mutation. The number of mutations per division has been set to reproduce the experimentally observed mutation frequency per daughter cell of 2/3 in the LAST model. Each mutation corresponds to a shift in the shape space to a von Neumann neighbor in a random direction.

Each seeder cell is assigned a unique random nucleotide sequence of 300 base pairs, representing the Ighv domain of a real BCR sequence. Due to restricting the modeled sequence to the Ighv domain, mutations in the shape space correspond to a random nucleotide replacement in the Ighv sequence only with a by 50% reduced mutation frequency.

After the required number of divisions the BC differentiates with a rate of in 1/6 minutes to the LZ phenotype. All BCs that kept the antigen up to this time, differentiate to output cells, up-regulate CXCR4, and leave the GC in direction of the T zone.

1.0.7 LZ B cell selection

At the time $t = 0$ of differentiation from the DZ to the LZ phenotype, BCs are in state *unselected* and BC-specific factors are initialized to

$$\begin{aligned} F(t=0) &= F_0 = 1 \\ C(t=0) &= C_0 = 0 \\ R(t=0) &= R_0 = 0 \end{aligned} \tag{2}$$

These factors are associated with FoxO1 (F), c-Myc (C), and mTOR (R) in the model. In BCs not in contact to Tfh, FoxO1, mTOR and c-Myc evolve according to

$$\frac{dF}{dt} = -\alpha_{\mathrm{F}} H_{\mathrm{F}}(B) F \tag{3}$$

$$\frac{dR}{dt} = \beta_{\mathrm{R}} H_{\mathrm{R}}(B) \tag{4}$$

$$\frac{dC}{dt} = -\gamma_{\mathrm{C}} C \quad , \tag{5}$$

with $\beta_{\mathrm{R}} = 1/(8\,\text{hours})$ the mTOR production rate modulated by the BCR-dependent Hill-function $H_{\mathrm{R}}(B)$ with K-value of 20 and Hill-coefficient 2. $\alpha_{\mathrm{F}} = 2/\text{hours}$ is the FoxO1 reduction rate modulated by the BCR-dependent Hill-function $H_{\mathrm{F}}(B)$ between 0 and 1 with K-value of 20 and Hill-coefficient 1. $\gamma_{\mathrm{C}} = \ln(2)/(3\,\text{hours})$ is the c-Myc degradation rate. R and C have different dynamics in state *Tfh-contact.*

LZ BCs can be in the states *unselected, FDC-contact, FDC-selected, Tfh-contact, selected, apoptotic.*

1.1 Unselected

LZ BCs migrate and search for contact with FDCs loaded with antigen in order to collect antigen for 0.7 hours. If an FDC soma or dendrite is present at the position of the BC, the BC attempts to establish contact to the epitope with highest affinity to the BCR (default setting). Alternatively, the BC may attempt to establish contact to the epitope of highest availability at this site. In both settings, binding is affinity dependent and happens with the probability b in Eq. (1). If the available number of antigen portions at the specific FDC site drops below 20 the binding probability b is linearly reduced with the number of available portions. If successful, the BC switches to the state *FDC-contact*; otherwise the BC continues to migrate. Further binding-attempts are prohibited for 1.2 minutes. At the end of the antigen collection period, BCs switch to the state *FDC-selected.* If a LZ BC fails to collect any antigen until this time it switches to the state *apoptotic.*

1.2 FDC-contact

LZ BCs remain immobile (bound) for 3 minutes [10] and then return to the state *unselected* or *FDC-selected*, depending on from which state contact to FDC was established. The counter for the number of successful antigen uptake events p and the degree of BCR-signaling B are increased by one unit and the FDC reduces its locally available antigen portions by one. Also, BC-sepcific factors mentiond before depend on the amount of presented pMHC p and the degree of BCR-signaling B.

1.3 FDC-selected

BCs search for contact with Tfh or FDC. If they meet a Tfh they switch to the state *Tfh-contact.* If they meet a FDC they switch to the state *FDC-contact* with probability b in Eq. (1). If Tfhs and FDCs are neighbors of a BC, the BC binds the Tfh.

1.4 Tfh-contact

LZ BCs remain immobile for a time determined by a Hill-function in dependence on the amount of presented pMHC p. This hill function has values between 0 and 0.2, K-value 15 and a Hill-coefficient of 2. During this time, the bound Tfh, which may also be bound to other BCs, polarizes to the BC with highest number of successful antigen uptakes. Polarization is determined from scratch in every time step and inertia from repolarization or intracellular organelles are ignored. If more than one BC shares the same highest pMHC presentation p, polarization is chosen randomly among those BCs. Only the BC to which the Tfh is polarized receives Tfh signals C and accumulate those.

Tfh signals are represented as mTOR levels as well as c-Myc levels, which are modulated by FoxO1, according to

$$\frac{dC}{dt} = \alpha_{\mathrm{C}}\left(1 - H_{\mathrm{C}}(F)\right) T(p) H_{\mathrm{I}}(t) \delta_{\mathrm{TB}} - \gamma_{\mathrm{C}} C \quad (6)$$

$$\frac{dR}{dt} = \alpha_{\mathrm{R}} H_{\mathrm{R}}(B) H_{\mathrm{I}}(t) T(p) \delta_{\mathrm{TB}} + \beta_{\mathrm{R}} H_{\mathrm{R}}(B) \quad . \quad (7)$$

$T(p)$ is the pMHC-dependent intensity of Tfh signaling to the interacting BC associated with CD40-signals, which is described by a Hill-function with values between zero and three, K-value 15 and a Hill-coefficient of 2. Signaling intensity is further modulated by the degree of ICOSL upregulation $H_{\mathrm{I}}(t)$. $H_{\mathrm{I}}(t)$ is a Hill-function in time with values between zero and one, determined by a characteristic ICOSL-upregulation time of four hours in mice [11] and Hill-coefficient two. α_{C} and γ_{C} are the growth and degradation rates of c-Myc. $\beta_{\mathrm{R}} = 1/(8\,\mathrm{hours})$ tunes the level of mTOR upregulation when BCs are not in contact to Tfh (see Eq. (4)), while $\alpha_{\mathrm{R}} = 1/(\mathrm{hours})$ determines mTOR upregulation during productive contact with Tfh. $H_{\mathrm{R}}(B)$ is a Hill-function describing the dependence of mTOR regulation on signals downstream of BCR. $H_{\mathrm{C}}(F)$ is a Hill-function controlling the suppression of c-Myc signals by FoxO1 with values between zero and one, K-value of 0.5 and Hill-coefficient of 1.

After the binding time, the BC detaches and returns to the state *FDC-selected*. It continues to search for and bind FDC or Tfhs, where binding twice the same Tfh in a sequence is excluded. The total period of signal acquisition in the LZ is finished when the LZ passage time gets longer than the number of antigen uptake events times 0.5 hours. When this limit is reached during a running contact, the contact is kept active until the end of the contact time. Then, it switches to the state *apoptotic* if either $R < 1$ or if the received Tfh-signals are $C < 0.3$. It switches to the state *selected* otherwise.

1.5 Selected

LZ BCs keep the LZ phenotype for 6 hours and desensitize for CXCL13, thus, perform a random walk. During that time they re-enter cell cycle and progress through the cell cycle phases. Then they recycle back to the DZ phenotype with a rate of 1/6 minutes and memorize the amount of collected antigen as well as the cell cycle phase they have achieved by this time.

The number of divisions $P(C)$ the recycled BCs will do is derived from the C, which reflects the c-Myc level, according to

$$P(C) \quad = \quad \frac{\gamma(\alpha, \beta C)}{\Gamma(\alpha)} \cdot r + 1 \,. \tag{8}$$

where r is a rescaling factor, α and β are the shape and scale parameters of the incomplete gamma function given by

$$\gamma(\alpha, \beta C) = \int_{\beta C}^{\infty} t^{\alpha-1} e^{-t} dt \tag{9}$$

To fit the experimental observations we put r to 31, α to 5 and β to 0.4. Reproducing an average number of two divisions per dark zone passage and a maximum number of divisions $>= 10$ for the observed c-Myc levels.

1.6 Apoptotic

LZ BCs remain on the lattice for 6 hours before they are deleted. They continue to be sensitive to CXCL13 during this time.

1.6.1 Chemokine distribution

Two chemokines CXCL12 and CXCL13 are considered. CXCL13 is produced by FDCs in the LZ with 10nMol per hour and FDC while CXCL12 is produced by stromal cells in the DZ with 400nMol per hour and stromal cell. As both cell types are assumed to be immobile, chemokine distributions were pre-calculated once and the resulting steady state distributions were used in all simulations.

1.6.2 Chemotaxis

DZ and LZ BCs regulate their sensitivity to CXCL13 and CXCL12, respectively. This is true in all BC states unless stated otherwise. All BCs move with a target speed of $7.5\mu m/min$. This leads to a slightly lower observable average speed of $\approx 6\mu m/min$.

BCs have a polarity vector that determines their preferential direction of migration. The polarity vector $\vec{p}$ is reset every 1.5 minutes into a new direction using the chemokine distribution c as

$$\vec{p} \quad = \quad \vec{p}_{\text{rand}} + \frac{\alpha}{1 + \exp\left\{\kappa\left(K_{1/2} - \Delta x |\vec{\nabla} c|\right)\right\}} \frac{\vec{\nabla} c}{|\vec{\nabla} c|} \quad , \tag{10}$$

where $\vec{p}_{\text{rand}}$ is a random polarity vector and the turning angle is sampled from the measured turning angle distribution ([12] Fig. S1B). $\alpha = 3$ determines the relative weight of the chemotaxis and random walk, $K_{1/2} = 2 \cdot 10^{11}$ Mol determines the gradient of half maximum chemotaxis weight, and $\kappa = 10^{10}$/Mol determines the steepness of the weight increase.

BCs de- and re-sensitize for their respective chemokine depending on the local chemokine concentration: The desensitization threshold is set to 4.5nMol and 0.08nMol for CXCL12 and CXCL13, respectively, which avoids cell clustering in the center of the zones. The resensitization threshold is set at 2/3 and 3/4 of the desensitization threshold for CXCL12 and CXCL13, respectively.

BCs can only migrate if the target node is free. If occupied and the neighbor cell is to migrate in the opposite direction (negative scalar product of the polarity vectors) both cells are exchanged with a probability of 0.5. This exchange algorithm avoids lattice artifacts leading to cell clusters.

Tfh do random walk with a preferential directionality to the LZ: The polarity vector $\vec{p}$ of Tfh is determined from a mixture of random walk $\vec{r}$ and the direction of the LZ $\vec{n}$ by

$$\vec{p} \quad = \quad (1-\alpha')\vec{r} + \alpha'\vec{n} \; , \tag{11}$$

where $\alpha' = 0.1$ is the weight of chemotaxis. This weight leads to a dominance of random walk with a tendency to accumulate in the LZ as found in experiment. TCs migrate with an average speed of $10\mu m/min$ and repolarize every 1.7 minutes [13].

Output cell motility is derived from plasma cell motility data to 3μm per minute with a persistence time of 0.75 minutes [12].

1.7 QUANTIFICATION AND STATISTICAL ANALYSIS

All graphs were generated with pythons matplotlib. The corresponding python files are part of the data at Zenodo. All simulations were repeated 1000 times with random number generator seeded by the CPU time. Mean and standard deviation are shown in the figures.

References

[1] Meyer-Hermann M, Mohr E, Pelletier N, Zhang Y, Victora GD, Toellner KM. A theory of germinal center B cell selection, division, and exit. Cell reports. 2012;2(1):162-74.

[2] Meyer-Hermann M. Overcoming the dichotomy of quantity and quality in antibody responses. The Journal of Immunology. 2014;193(11):5414-9.

[3] Binder SC, Meyer-Hermann M. Implications of intravital imaging of murine germinal centers on the control of B cell selection and division. Frontiers in immunology. 2016;7:593.

[4] Meyer-Hermann M. Injection of antibodies against immunodominant epitopes tunes germinal centers to generate broadly neutralizing antibodies. Cell reports. 2019;29(5):1066-73.

[5] Meyer-Hermann M. A molecular theory of germinal center B cell selection and division. Cell Reports. 2021;36(8).

[6] Perelson AS, Oster GF. Theoretical studies of clonal selection: minimal antibody repertoire size and reliability of self-non-self discrimination. Journal of theoretical biology. 1979;81(4):645-70.

[7] Meyer-Hermann M, Deutsch A, Or-Guil M. Recycling probability and dynamical properties of germinal center reactions. Journal of Theoretical Biology. 2001;210(3):265-85.

[8] Victora GD, Schwickert TA, Fooksman DR, Kamphorst AO, Meyer-Hermann M, Dustin ML, et al. Germinal center dynamics revealed by multiphoton microscopy with a photoactivatable fluorescent reporter. Cell. 2010;143(4):592-605.

[9] Gitlin AD, Shulman Z, Nussenzweig MC. Clonal selection in the germinal centre by regulated proliferation and hypermutation. Nature. 2014;509(7502):637-40.

[10] Schwickert TA, Lindquist RL, Shakhar G, Livshits G, Skokos D, Kosco-Vilbois MH, et al. In vivo imaging of germinal centres reveals a dynamic open structure. Nature. 2007;446(7131):83-7.

[11] Papa I, Saliba D, Ponzoni M, Bustamante S, Canete PF, Gonzalez-Figueroa P, et al. TFH-derived dopamine accelerates productive synapses in germinal centres. Nature. 2017;547(7663):318-23.

[12] Allen CD, Okada T, Tang HL, Cyster JG. Imaging of germinal center selection events during affinity maturation. Science. 2007;315(5811):528-31.

[13] Miller MJ, Wei SH, Parker I, Cahalan MD. Two-photon imaging of lymphocyte motility and antigen response in intact lymph node. Science. 2002;296(5574):1869-73.
