# Supplementary material for: Transient silencing of hypermutation preserves B cell affinity during clonal bursting
Source: Nature. 2025 Mar 19;641(8062):486–94. doi: 10.1038/s41586-025-08687-8 (PMC12058519; doi:10.1038/s41586-025-08687-8)
Supplement: Supplementary file 2 — Reporting Summary [file 41586_2025_8687_MOESM2_ESM.pdf]

nature portfolio

Corresponding author(s): G.D.Victora
Last updated by author(s): 11/19/2024

Reporting Summary

Nature Portfolio wishes to improve the reproducibility of the work that we publish. This form provides structure for consistency and transparency in reporting. For further information on Nature Portfolio policies, see our Editorial Policies and the Editorial Policy Checklist.

Statistics

For all statistical analyses, confirm that the following items are present in the figure legend, table legend, main text, or Methods section.

n/a	Confirmed	
☐	☒	The exact sample size (*n*) for each experimental group/condition, given as a discrete number and unit of measurement
☐	☒	A statement on whether measurements were taken from distinct samples or whether the same sample was measured repeatedly
☐	☒	The statistical test(s) used AND whether they are one- or two-sided<br>*Only common tests should be described solely by name; describe more complex techniques in the Methods section.*
☐	☒	A description of all covariates tested
☐	☒	A description of any assumptions or corrections, such as tests of normality and adjustment for multiple comparisons
☐	☒	A full description of the statistical parameters including central tendency (e.g. means) or other basic estimates (e.g. regression coefficient) AND variation (e.g. standard deviation) or associated estimates of uncertainty (e.g. confidence intervals)
☐	☒	For null hypothesis testing, the test statistic (e.g. *F*, *t*, *r*) with confidence intervals, effect sizes, degrees of freedom and *P* value noted<br>*Give P values as exact values whenever suitable.*
☒	☐	For Bayesian analysis, information on the choice of priors and Markov chain Monte Carlo settings
☒	☐	For hierarchical and complex designs, identification of the appropriate level for tests and full reporting of outcomes
☒	☐	Estimates of effect sizes (e.g. Cohen's *d*, Pearson's *r*), indicating how they were calculated

Our web collection on statistics for biologists contains articles on many of the points above.

Software and code

Policy information about availability of computer code

Data collection	Data collection code was not used in this study
Data analysis	Graphs were plotted using R (v.4.3.1), and formatted in Adobe Illustrator CS. Statistical tests were performed in R (v.4.3.1). Flow cytometry data was analyzed using FlowJo v10. Computational analysis of single-cell RNA sequencing data used CellRanger v6.0.1, v7.0.1, and v8.0.1; and R v. v.4.3.1. Code implementing simulations of clonal burst birth-death processes used in Figure 1 is available at https://github.com/WSDeWitt/aid-sim. Full description of Agent-Based GC Simulation Model used in Figure 4 is provided in the Supplementary Text. Igh sequencing analysis was carried out using PANDASeq v.2.11, HighVQUEST v. 1.6.9, and GCtree (deWitt et. al. 2018).

For manuscripts utilizing custom algorithms or software that are central to the research but not yet described in published literature, software must be made available to editors and reviewers. We strongly encourage code deposition in a community repository (e.g. GitHub). See the Nature Portfolio guidelines for submitting code & software for further information.

Data

Policy information about availability of data

All manuscripts must include a data availability statement. This statement should provide the following information, where applicable:

- Accession codes, unique identifiers, or web links for publicly available datasets
- A description of any restrictions on data availability
- For clinical datasets or third party data, please ensure that the statement adheres to our policy

All raw Ig sequencing data generated for this study will be promptly available for the community upon publication

Research involving human participants, their data, or biological material

Policy information about studies with human participants or human data. See also policy information about sex, gender (identity/presentation), and sexual orientation and race, ethnicity and racism.

Reporting on sex and gender	N/A
Reporting on race, ethnicity, or other socially relevant groupings	N/A
Population characteristics	N/A
Recruitment	N/A
Ethics oversight	N/A

Note that full information on the approval of the study protocol must also be provided in the manuscript.

Field-specific reporting

Please select the one below that is the best fit for your research. If you are not sure, read the appropriate sections before making your selection.

☒ Life sciences ☐ Behavioural & social sciences ☐ Ecological, evolutionary & environmental sciences

For a reference copy of the document with all sections, see nature.com/documents/nr-reporting-summary-flat.pdf

Life sciences study design

All studies must disclose on these points even when the disclosure is negative.

Sample size	No statistical methods were used to determine sample size. Numbers of mice per group within each independent experiment were limited to numbers typically used in the field. The cell line experiment was performed twice with triplicate technical replicates, as is the standard for such assays.
Data exclusions	For 10x data presented in Fig. 4, a sample with small GC size was collected but excluded for analysis.
Replication	Experiments were performed multiple times independently, as described in the figure legends.
Randomization	Littermate mice were used to control for litter, cage, and age effects. Mice were divided stochastically (albeit without a specific randomization procedure) between experimental groups.
Blinding	Experimenters were not blinded to experimental group, since most readouts (e.g. FACS fluorescence intensities) are not subjective.

Reporting for specific materials, systems and methods

We require information from authors about some types of materials, experimental systems and methods used in many studies. Here, indicate whether each material, system or method listed is relevant to your study. If you are not sure if a list item applies to your research, read the appropriate section before selecting a response.

Materials & experimental systems

n/a	Involved in the study	
☐	☒	Antibodies
☐	☒	Eukaryotic cell lines
☒	☐	Palaeontology and archaeology
☐	☒	Animals and other organisms
☒	☐	Clinical data
☒	☐	Dual use research of concern
☒	☐	Plants

Methods

n/a	Involved in the study	
☒	☐	ChIP-seq
☐	☒	Flow cytometry
☒	☐	MRI-based neuroimaging

Antibodies

Antibodies used	See Extended Data Table 1.
Validation	All antibodies validated on the manufacturers' websites.

Eukaryotic cell lines

Policy information about cell lines and Sex and Gender in Research

Cell line source(s)	NB-21.2D9 feeder cells were provided by G. Kelsoe (Duke University)
Authentication	Cell lines were used solely for the purpose of maintaining GC B cells in culture, which were confirmed successful as per the experimental data. No further authentication was performed beyond that provided by the original source.
Mycoplasma contamination	Cell lines were not tested after receipt from the original source but are were used at low passage numbers (3-4 passages).
Commonly misidentified lines (See ICLAC register)	No commonly misidentified cell lines were used.

Animals and other research organisms

Policy information about studies involving animals; ARRIVE guidelines recommended for reporting animal research, and Sex and Gender in Research

Laboratory animals	5-12 week old adult male and female mice on the C57BL/6J background were used. See 'mice' section in the methods for further details. Mice were housed at 72 °F (22.2 °C) and 30–70% humidity in a 12-h light/dark cycle with ad libitum access to food and water.
Wild animals	The study did not involve wild animals.
Reporting on sex	Both sexes of mice were used throughout the study. No significant differences were noted between sexes.
Field-collected samples	The study did not involve samples collected from the field.
Ethics oversight	All protocols were approved by the Rockefeller University Institutional Animal Care and Use Committees (protocol number 22058-H).

Note that full information on the approval of the study protocol must also be provided in the manuscript.

Plants

Seed stocks	*Report on the source of all seed stocks or other plant material used. If applicable, state the seed stock centre and catalogue number. If plant specimens were collected from the field, describe the collection location, date and sampling procedures.*
Novel plant genotypes	*Describe the methods by which all novel plant genotypes were produced. This includes those generated by transgenic approaches, gene editing, chemical/radiation-based mutagenesis and hybridization. For transgenic lines, describe the transformation method, the number of independent lines analyzed and the generation upon which experiments were performed. For gene-edited lines, describe the editor used, the endogenous sequence targeted for editing, the targeting guide RNA sequence (if applicable) and how the editor was applied.*
Authentication	*Describe any authentication procedures for each seed stock used or novel genotype generated. Describe any experiments used to assess the effect of a mutation and, where applicable, how potential secondary effects (e.g. second site T-DNA insertions, mosiacism, off-target gene editing) were examined.*

Flow Cytometry

Plots

Confirm that:

- [x] The axis labels state the marker and fluorochrome used (e.g. CD4-FITC).
- [x] The axis scales are clearly visible. Include numbers along axes only for bottom left plot of group (a 'group' is an analysis of identical markers).
- [ ] All plots are contour plots with outliers or pseudocolor plots.
- [x] A numerical value for number of cells or percentage (with statistics) is provided.

Methodology

Sample preparation	Cells were isolated from lymph nodes by maceration with disposable micropestles (Axygen) in 100 µl of PBS supplemented with 0.5% BSA and 2mM EDTA (PBE), and single cell suspensions obtained by two passes through a 70 µm mesh. Cells were stained with fluorescently labeled antibodies on ice for 30 minutes. See methods for further details.
Instrument	BD FACSSymphony A5 for flow cytometry, BD FACSymphony S6 for cell sorting, BD FACSDiscover S8 for image-based sorting
Software	FlowJo v.10 software
Cell population abundance	Cell sorting was performed for Chromium single cell RNA sequencing. Cell population abundance was confirmed by quantification of hashtag oligos used to identify different samples and cell types.
Gating strategy	All positive and negative populations were determined by compensation with single color controls. For sorting and analysis, all lymphocytes were first gated based on SSC-A vs FSC-A, followed by 2 singlet gates (FSC-H vs FSC-A and SSC-H vs SSC-A). For GC gating, cells were gated on B220+, TCRb-, CD38-, and Fas+. Gating strategy for image-based cell sorting is shown in Extended Data Figure 6a.

- [x] Tick this box to confirm that a figure exemplifying the gating strategy is provided in the Supplementary Information.
